# Conserved Asp233–Asp246 hydrogen bond modulates active site dynamics in class A *β*‐lactamases

**DOI:** 10.1002/pro.70798

**Published:** 2026-09-17

**Authors:** Cristina V. Lopez‐Gallego, Monika Timmer, Jocelyne Vreede, Marcellus Ubbink

**Affiliations:** ^1^ Leiden Institute of Chemistry Leiden University Leiden The Netherlands; ^2^ Van 't Hoff Institute of Molecular Sciences University of Amsterdam Amsterdam The Netherlands

**Keywords:** active site, biocatalysis, BlaC, conserved residues, molecular dynamics, protein dynamics, substrate specificity, *β*‐lactamase

## Abstract

Rigid enzymes catalyze chemical reactions by stabilizing the transition state through a specific conformation. Catalytic residues are precisely positioned, and enzyme dynamics are kept to a minimum. It is thought that conserved residues around the active site (second‐shell residues) play a crucial role in positioning catalytic residues. Asp233 and Asp246 are two highly conserved second‐shell residues in class A β‐lactamases, rigid enzymes that inactivate *β*‐lactam antibiotics. The two aspartates share a short hydrogen bond, linking β‐strands 3 and 4. The role of this interaction in *Mycobacterium tuberculosis* β‐lactamase BlaC was studied by mutating the Asp residues to Ala. Disruption of the hydrogen bond subtly affects the activity and stability of the enzyme, suggesting the interaction helps to fine‐tune the active site. The effects are larger for BlaC D246A than for D233A, indicating that effects cannot solely be attributed to the loss of the hydrogen bond. Molecular dynamics calculations indicate a shift in the conformational landscape due to the mutations, altering the conformational equilibria of the catalytic residues toward less active states. The results illustrate that second‐shell residues act as a complex network that supports the efficient positioning of the catalytic residues.

## 1 INTRODUCTION

Enzymes are three‐dimensional structures with a precise spatial arrangement that enables catalysis of chemical reactions with exceptional selectivity and efficiency. The catalytic power of enzymes arises from multiple complementary mechanisms. Their ability to stabilize the transition state plays a key role, lowering the energy barrier for product formation. Through evolutionary optimization, enzymes have adopted conformations that achieve an optimal balance between activity and selectivity. In complex chemical reactions, coordinated and specific motions are often required for optimal function (Crean et al., 2020; James & Tawfik, 2003; Tokuriki & Tawfik, 2009; Wei et al., 2016), whereas rigid enzymes exist predominantly in a single, well‐defined conformation. The finely tuned geometries in combination with minimal dynamics are central for substrate specificity. The enzyme‐substrate interactions are delicate; sometimes even a single amino acid substitution can shift specificity toward an alternative substrate (Knoverek et al., 2021; Sikosek et al., 2016; Soroka et al., 2017). The residues directly surrounding the active site, the second shell, are thought to fine‐tune the positioning of the active site residues, whereas residues distal from the active site, the third shell, are primarily involved in maintaining the overall structure of the enzyme (Chikunova & Ubbink, 2022). The functional importance of second‐shell residues in enzyme catalysis has been demonstrated across multiple systems. For example, in phosphate‐irrepressible alkaline phosphatase from *Flavobacterium* (PafA), MD simulations combined with experimental validation revealed that the second shell contributes to pre‐organizing the resting state of the enzyme, thereby influencing substrate binding (Deng & Cui, 2023). In superoxide dismutase MnSOD, the conserved residue Gln146 was proven to have a role in fine‐tuning the redox‐active site and ensuring proper metal loading and enzymatic function (Miller & Wang, 2017). In adenosine kinase from *Leishmania donovani*, the residue Gly62, located near the active site, provides the flexibility required for the conformational changes necessary for substrate binding (Datta et al., 2005). The conserved residue Glu50 was shown to play a critical role in maintaining the structural integrity, stability, and function of 1‐Cys peroxiredoxin 6 (Prdx6) (Qausain et al., 2023). Other studies have highlighted important second‐shell residues and interactions in *β*‐lactamases. Point mutations at second‐shell positions in PenA have shown to alter substrate specificity, such as for residues 69, 136, 169, and 170 (Yi et al., 2012). TEM‐1 *β*‐lactamase can tolerate multiple second‐shell substitutions, most notably G238S, often in combination with other mutations, leading to an expanded substrate spectrum (de Wals et al., 2009). In a systematic mutagenesis effort of BlaC from *Mycobacterium tuberculosis* by our group, the roles of conserved non‐catalytic residues were analyzed, leading to the conclusion that conserved second‐shell residues fine‐tune active‐site geometry. Mutations of these residues yielded correctly folded but catalytically impaired proteins, whereas more distant, third‐shell residues mainly affect folding/aggregation (Chikunova & Ubbink, 2022). Mutations in second‐shell residues generally result in small structural effects (as probed by NMR spectroscopy) over the entire active site region, indicative of the interrelationship of residues in and around the active site. For third‐shell residues, mutation effects were more local (Chikunova & Ubbink, 2022). For example, the *cis* peptide bond between Glu166 and Pro167 in BlaC was demonstrated to be important for maintaining the Ω‐loop in its native conformation. Mutation P167S converts the peptide bond to *trans* and enlarges the active site pocket, causing a shift in substrate specificity (Sun et al., 2023). Also, changes at position 105 in class A *β*‐lactamases result in an enlarged and more flexible active site, promoting inhibitor resistance (Feiler et al., 2013; Li et al., 2012; Papp‐Wallace et al., 2010; Radojković et al., 2025).

Class A serine *β*‐lactamases are bacterial enzymes that inactivate *β*‐lactam antibiotics and represent one of the most common mechanisms of antibiotic resistance (AA., 1984). This class of enzymes serves as a good model for studying how evolution shapes the active site of enzymes, because *β*‐lactamases are amenable to straightforward laboratory evolution experiments (Petrosino et al., 1998). The active site comprises eight highly conserved residues involved in catalysis and substrate recognition (Ser70, Lys73, Ser130, Glu166, Lys234, Thr235, Gly236, and Thr237, based on Ambler numbering (Ambler et al., 1991; Papp‐Wallace et al., 2010; Radojković et al., 2025)). Ser70 forms a covalent bond with the substrate during the catalytic reaction; Lys73 acts as a general base for the acylation reaction; Ser130 serves as a proton donor during the hydrolysis; and Glu166 promotes the deacylation step by activation of a water molecule (Lewandowski et al., 2018; Meroueh et al., 2005; Minasov et al., 2002; Pemberton et al., 2020). The KTGT motif (Lys234, Thr235, Gly236, and Thr237) is involved in substrate recognition, contributing to substrate binding through interactions with the carboxyl group on *β*‐lactam antibiotics (Delmas et al., 2010; Golemi‐Kotra et al., 2004; Jeon et al., 2015).

**FIGURE 1:**
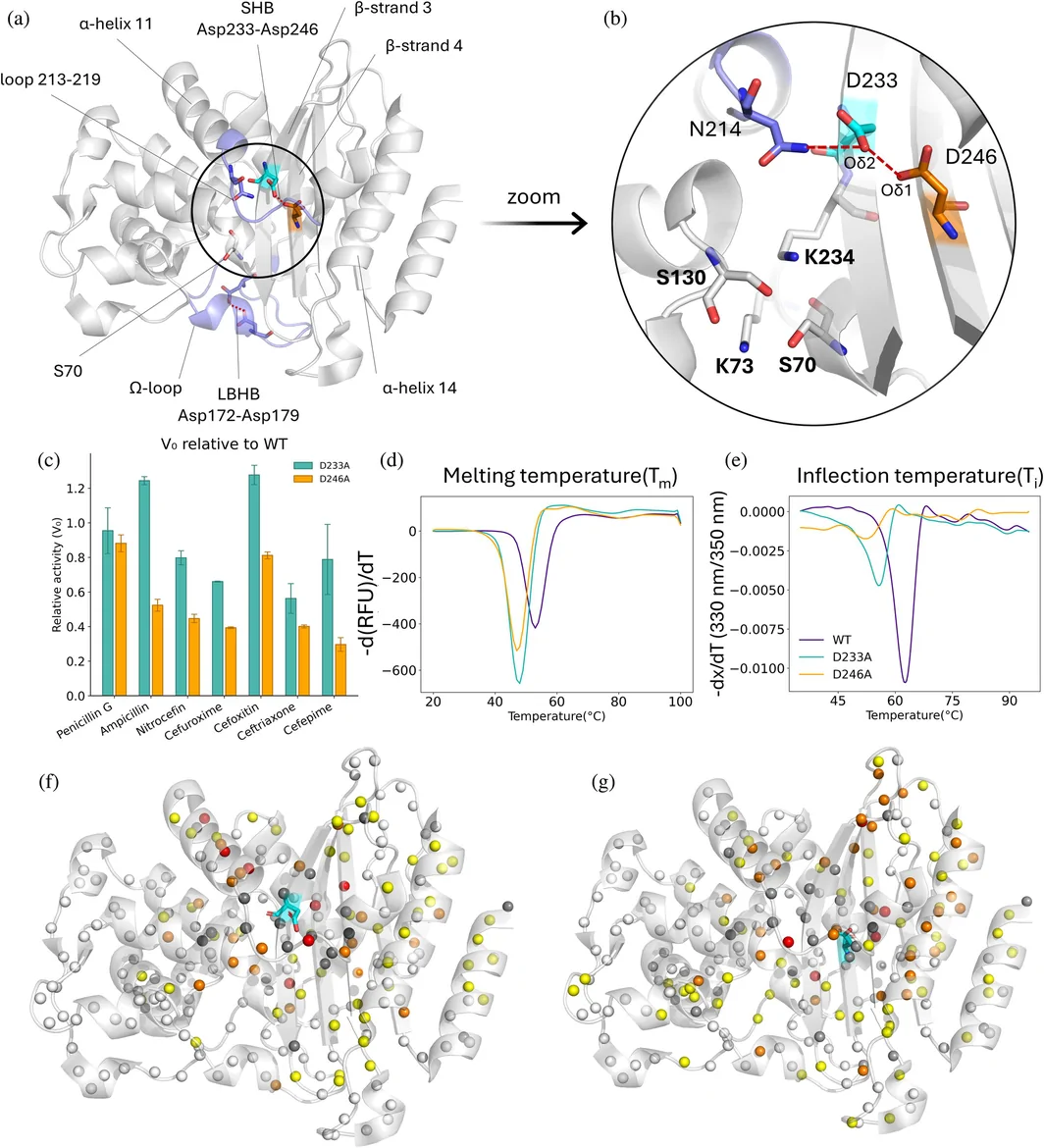
Activity, stability, and chemical shift perturbations (CSPs) of BlaC D233A and D246A. (a) Cartoon representation of BlaC illustrating its overall fold (PDB ID: pdb_00002gdn (Wang et al., 2006)). Functionally important loop regions are highlighted in slate. Residues Ser70, Asp172, Asp179, Asn214, Asp233 (cyan), and Asp246 (orange) are displayed in stick representation to indicate their positions in the protein. The black circle highlights the area is magnified in (b). (b) Enlarged view of the crystal structure. Residues Ser70, Lys73, Ser130, Asn214 (slate), Asp233 (cyan), Lys234, and Asp246 (orange) are represented in sticks, active site residues are labeled in bold. (c) Initial velocities (*V*_0_) for hydrolysis of various substrates relative to BlaC WT. The error bars represent the standard deviation of three replicates. (d) Negative derivative of the relative fluorescence units (RFU) signal from thermal shift assay. The curve is averaged across three experiments, and the standard deviation of the absolute minima for the WT, D233A, and D246A variants is 0.5°C, for legend, see panel (e). (e) Negative derivative of the ratio of the fluorescence signals at 350 and 330 nm to determine the inflection temperature. The curve is averaged over three experiments, and the standard deviations of the absolute minima for BlaC WT, D233A, and D246A are 0.06, 0.2, and 0.2°C, respectively. (f), (g) CSPs for BlaC D233A (f) and D246A (g), compared to the WT amide resonances. Spectrum were recorded at pH 6.4 in 100 mM phosphate buffer at 25°C. The mutation site is shown in cyan sticks, and backbone amides are indicated as spheres, colored by CSP. White: CSP< 0.025 ppm, yellow: 0.025< CSP< 0.05 ppm, orange: 0.05< CSP< 0.1 ppm, red: CSP> 0.1 ppm, gray: No data available.

We demonstrated before the importance of the low‐barrier hydrogen bond (LBHB) between Asp172 and Asp179 in a region named the Ω‐loop for substrate specificity of BlaC (Sun et al., 2024). It was found that deprotonation occurs with a p*K*_a_ between 9 and 10. LBHBs are a special type of hydrogen bond that is shorter than normal H‐bonds and has a proton equally shared between the donor and acceptor. These interactions are generally considered to be stronger than a conventional HB; however, its existence and relevance have been the subject of extensive debates (Kemp, Lewandowski, & Chen, 2021). This interaction, present between both catalytic and non‐catalytic residues (Hosur et al., 2013), has been proposed to act as a proton‐relay station during catalysis, whereas in non‐catalytic residues it helps maintain the structure of the enzyme (Nichols et al., 2015). LBHBs are inherently difficult to characterize experimentally and computationally. Prior work by Kemp et al. in 2021 provided evidence through a subatomic resolution crystal structure of class A *β*‐lactamase CTX‐M, in which the hydrogen between Asp233Oδ1 and Asp246Oδ2 was equidistant to both oxygens, separated by 0.247 nm (Kemp, Nichols, et al., 2021). This distance aligns with the definition of a short hydrogen bond (SHB), and may be indicative of a LBHB. In the present study, we study this SHB in BlaC. No sub‐Ångström structure has been reported for the wild‐type (WT) enzyme. The distance between Asp233Oδ1 and Asp246Oδ2 is 0.25 nm, based on the pdb_00002gdn crystal structure (resolution 0.172 nm) (Wang et al., 2006). This separation falls within the SHB range, but as it is not established to be a LBHB, it is referred to as a SHB in this work. The SHB between residues Asp233 and Asp246 is located in the second‐shell and is therefore hypothesized to contribute to the precise positioning of catalytic residues. The residues Asp233 and Asp246 are located in *β*‐strands 3 and 4, respectively. In addition to interacting with Asp246, Asp233 forms a hydrogen bond with Asn214, which is located in the loop 213–220 (Figure 1a,b). The C*α* atoms are 0.9 nm (Asp233) and 1.1 nm (Asp246) from that of Ser70. Both residues are highly conserved, suggesting that they may have a general role in fine‐tuning the active sites of *β*‐lactamases (Chikunova & Ubbink, 2022; Kemp, Nichols, et al., 2021).

We eliminated the SHB by mutation to Ala of either of the Asp residues. It was observed that the BlaC D233A and D246A are active but exhibit substrate specificity changes and a reduction in stability. The effects differ in magnitude between BlaC D233A and D246A, with the latter being more severe, indicating that the effects are not solely a consequence of eliminating the SHB. Both mutations cause widespread chemical shift perturbations (CSPs) of the amides, in line with previous results for mutations of second‐shell residues (Chikunova & Ubbink, 2022). Molecular dynamics (MD) simulations show that the mutations cause shifts in conformational equilibria of active site residues. In combination, the results provide an example of how second‐shell residues may affect the precise positioning of active site residues by subtle changes in conformational equilibria.

## 2 RESULTS

### 2.1 The disruption of the SHB between Asp233 and Asp246 impacts the activity and thermal stability of BlaC

To understand the contribution of the interaction between Asp233 and Asp246 to the activity of BlaC, the mutations D233A and D246A were introduced in the gene for BlaC and the mutant genes were overexpressed in *Escherichia coli*. The variants yielded folded and active enzymes. The kinetics of catalytic activity toward seven *β*‐lactam substrates were evaluated by measuring the initial reaction velocities (*V*₀). Full Michaelis–Menten curves were obtained for representative substrates (Figure S1, Table S1). The impact of the mutations on catalysis is subtle (Figure 1c) but the two mutations have distinct effects. In general, BlaC D233A has higher catalytic efficiency than BlaC D246A, and in two cases, higher than BlaC WT, and the change in activity differs between the two variants (Figure 1c). This difference indicates that the individual mutations have more effects than the mere removal of the SHB, which would be expected to produce the same effects in both mutants when the proton is shared equally between the two residues.

The thermostability of the purified BlaC D233A and D246A was measured by determining the melting point (*T*_m_) using an extrinsic fluorescence dye, and by the inflection temperature (*T*_i_) based on changes in the intrinsic tryptophan and tyrosine fluorescence. BlaC D233A and D246A showed melting points of 48 and 47°C, respectively, corresponding to decreases of 5 and 6°C compared to WT; thus, both variants are similarly stable at equilibrium conditions (Figure 1d). BlaC D233A and D246A showed inflection temperatures of 55.7 and 52.3°C, a reduction of 7.0 and 10.4°C, respectively, compared to WT (62.7°C). These large reductions in melting and inflection temperatures are quite remarkable, considering that the enzymes remain functional, at least at 25°C. *T*_m_ and *T*_i_ were obtained from the negative derivative of the sigmoidal curve, where the absolute minima represent the point with the highest rate of change. The derivative inflection profiles of BlaC D233A and D246A show less intense and broader peaks than those of BlaC WT, suggesting that the unfolding process is less cooperative, especially for BlaC D246A (Figure 1e).

### 2.2 BlaC D233A and D246A show chemical shift perturbations around the active site

To assess the structural effects of the Ala mutations, ^1^H–^15^N TROSY‐HSQC NMR spectra were recorded. CSPs were calculated for each amide to detect per‐residue chemical shift differences between the WT and the mutant. Extensive CSPs were observed for the BlaC variants compared to the WT spectrum (Figure S2). HNCA NMR spectra were obtained to assign the backbone amide resonances of the variants, in combination with the published assignments for BlaC WT (Elings et al., 2017). Both variants exhibit CSPs throughout a large part of the protein, particularly around the active site and in the *β*‐sheet, indicative of subtle but widespread structural rearrangements (Figure 1f,g). BlaC D246A generally shows stronger effects, as reflected in the CSPs mapped onto the structure, especially in helix‐11, helix‐14, and the loops that connect the *β*‐strands. The CSP‐maps for the two variants differ in many places, again indicative of effects that are not only a consequence of the removal of the SHB, which would be expected to produce very similar patterns.

### 2.3 WT molecular dynamics simulations show stable SHBs

**FIGURE 2:**
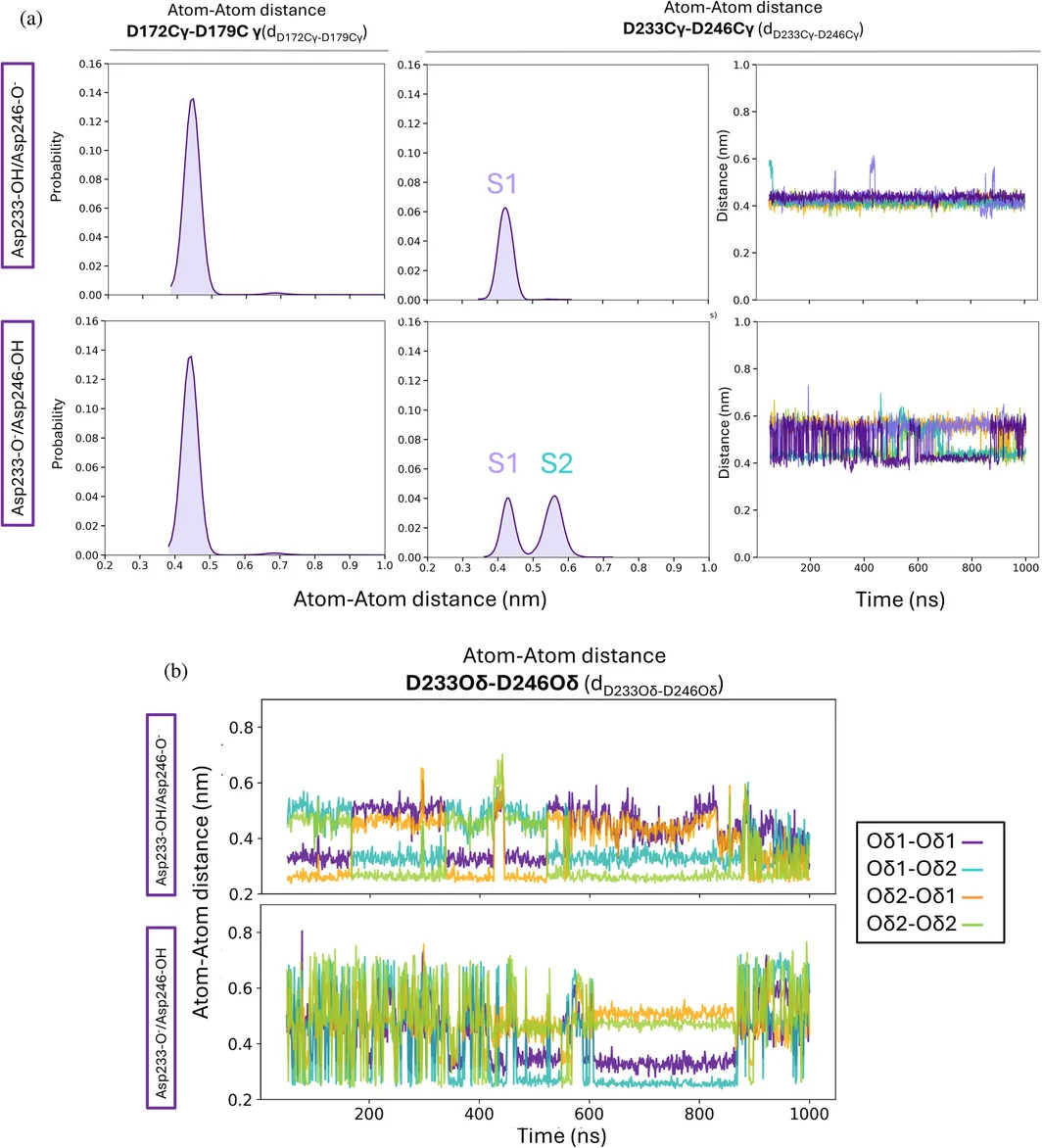
MD simulations results. (a) Probability distribution of the distance between the γ‐carbons of Asp172 and Asp179 (left) and the γ‐carbons of Asp233 and Asp246 (center) for BlaC WT in the Asp233‐OH/Asp246‐O^−^ and Asp233‐O^−^/Asp246‐OH simulations, indicating states S1 and S2. The distributions represent the probabilities over five replicates of 1 μs simulations. The time traces for the distance between the γ‐carbons of Asp233 and Asp246 are shown in the right panel; each color represents a different replicate. Kolmogorov–Smirnov tests of BlaC WT Asp233‐OH/Asp246‐O^−^ versus Asp233‐O^−^/Asp246‐OH show no significant difference for γ‐carbons Asp172 and Asp179, yielding *D* = 0.0269 and *p* = 0.064 and a significant difference for γ‐carbons of Asp233 and Asp246, yielding *D* = 0.571 and p<< 0.001. (b) Representative replicates of BlaC WT Asp233‐OH/Asp246‐O^−^ and Asp233‐O^−^/Asp246‐OH simulations showing the distance between carboxy oxygens of Asp233 and Asp246. The distance between Asp233Oδ1‐Asp246Oδ1 is represented in purple, Asp233Oδ1–Asp246Oδ2 in turquoise, Asp233Oδ2–Asp246Oδ1 in orange, and Asp233Oδ2–Asp246Oδ2 in pistachio. Note the correlated distance chances that reflect rotations around the χ_2_ dihedral angle(s).

To characterize the structural dynamics at atomic level, MD simulations were performed. First, we wanted to establish if the SHB was stable in a classic MD simulation in which the proton is assigned to one of the two Asp residues. Therefore, we tested two cases for WT BlaC, with either Asp233 or Asp246 protonated (denoted as Asp233‐O^−^/Asp246‐OH and Asp233‐OH/Asp246‐O^−^). Each simulation comprised five replicates of 1 μs each. To monitor the stability of SHBs, the interaction between Asp172 and Asp179 (d_D172Cγ‐D179Cγ_) was used as a control, as it is well‐established (Sun et al., 2024), with a separation of 0.43 nm between γ‐carbons in the crystal structure (pdb_00002gdn (Wang et al., 2006)). The distance distribution shows that the predominant distance between these two atoms is between 0.40 and 0.50 nm, resulting in an average of 0.442 (0.005) nm in both the Asp233‐O^−^/Asp246‐OH and Asp233‐OH/Asp246‐O^−^ simulations (Figure 2a, Figure S3, and Table S2). Thus, the Asp172–Asp179 H‐bond is stable in these simulations. Then, the distance between the γ‐carbons of Asp233 and Asp246 (d_D233Cγ–D246Cγ_) was assessed. In the crystal structure, this distance is 0.41 nm. Across all BlaC WT Asp233‐OH/Asp246‐O^−^ replicates, the distance remained between 0.40 and 0.45 nm, with a mean of 0.421 (0.009) nm (Figure S3, Table S2). The analysis of the four pair‐wise distances between the carboxy oxygens of Asp233 and Asp246 (d_D233Oδ–D246Oδ_) of the Asp233‐OH/Asp246‐O^−^ simulation showed that side‐chain flips over the χ_2_ dihedral angle occur frequently, but constantly two oxygen atoms remain at H‐bond distance (0.24–0.27 nm, Figures 2b, S4, and S5). These observations show that the interaction between Asp233 and Asp246 is stable throughout the simulation and the active site arrangement resembles the crystal structure (Figures 2b, S4). That was not the case in the simulation in which Asp246 is protonated (Asp233‐O^−^/Asp246‐OH). Here, the d_D233Cγ‐D246Cγ_ plot indicates that two states are sampled (Figure 2b). In state 1 (S1), the short distance between the Cγ atoms is maintained (d_D233Cγ–D246Cγ_ = 0.40–0.45) but Asp233, Ser130 and Lys234 are displaced, whereas state 2 (S2) (d_D233Cγ–D246Cγ_ = 0.52–0.59) the structure is more similar to the crystal structure, despite the loss of the SHB. Also the analysis of the carboxy oxygens atoms shows that the H‐bond is absent part of the time (Figure 2b). It is concluded that the combination of protonated Asp233 and deprotonated Asp246 best represents a stable WT protein. This is in line with findings of (Chikunova & Ubbink, 2022) who reported stability and catalytic performance for BlaC D233N similar to WT BlaC, and destabilization for BlaC D246N, suggesting that Asp233 has a stronger H‐bond donating character than Asp246 (Chikunova & Ubbink, 2022).

### 2.4 The conformation of catalytic residue Ser130 is affected by the D233A and D246A mutations

**FIGURE 3:**
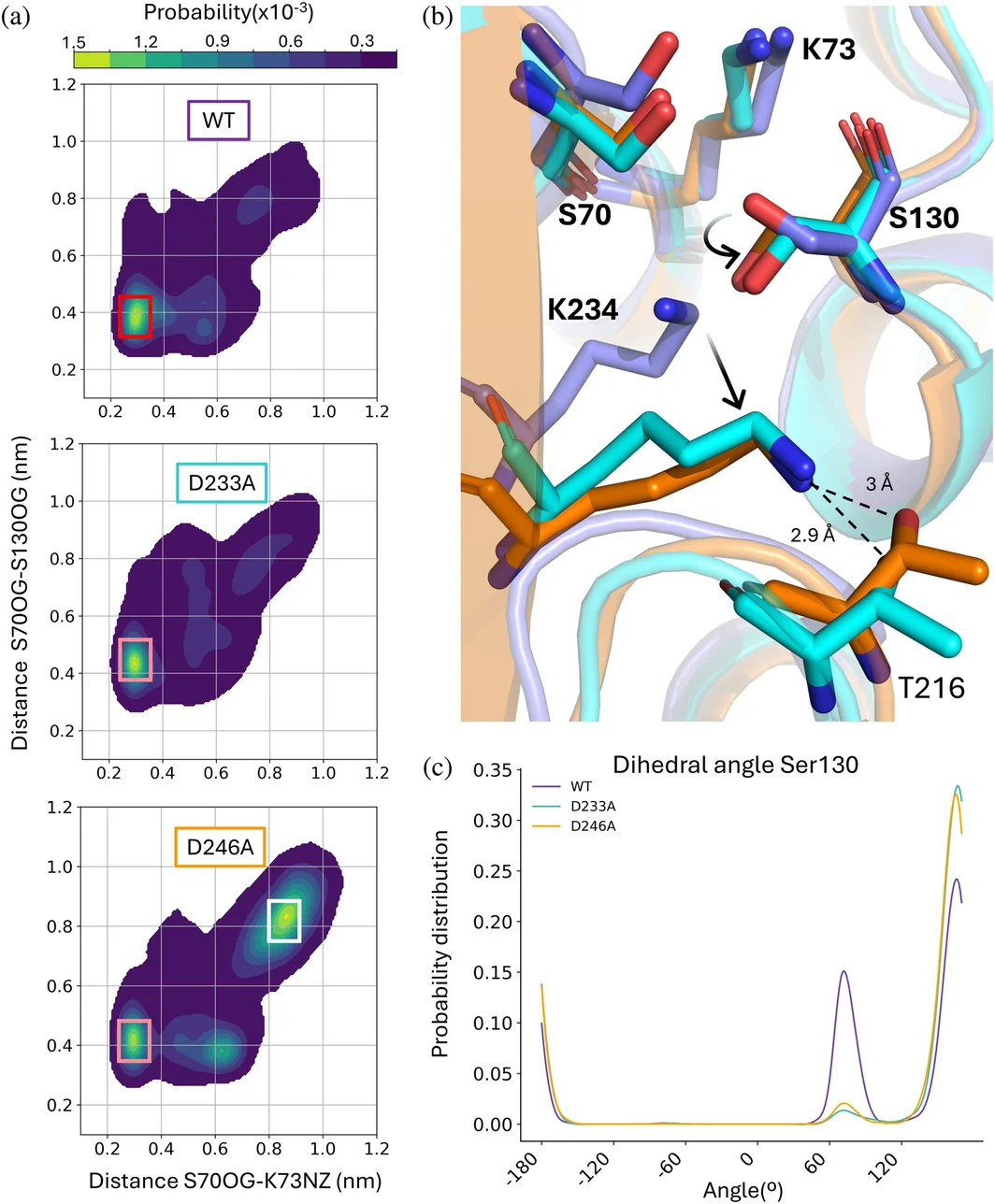
MD simulations reveal two conformations for Ser130. (a) Distance correlations between Ser70 Oγ and Lys73 Nζ atom (horizontal axis) and Ser70 Oγ and Ser130 Oγ (vertical axis) are plotted as probability densities. The rectangles highlight states A (red), A′ (salmon), and B (white). (b) States A and A′ sampled in MD simulations. The residues Ser70, Lys73, Ser130, Thr216, and Lys234 are shown as sticks, and the catalytic residues are indicated with labels in bold. The superimposed structures correspond to state A of BlaC WT (slate) (extracted from replicate 1 at 115 ns), state A′ of BlaC D233A (cyan) (replicate 3 at 94 ns), and state A′ of BlaC D246A (orange) (replicate 1 at 302 ns). The arrows highlight conformational changes in catalytic residues and the potential H‐bond between Lys234 and Thr216 is shown as a dashed line. (c) The probability distributions of the χ_1_ dihedral angle of S130 are shown. Probability distributions were calculated from five replicates of 1 μs MD simulations for the BlaC WT, D233A, and D246A. Kolmogorov–Smirnov tests show that the mutant distributions differ significantly from the WT distribution, yielding *D* = 0.245 for D233A and *D* = 0.237 for D246A (both *p*<< 0.001, Figure S9).

To probe the contribution of the interaction between Asp233 and Asp246 in positioning the catalytic residues, MD simulations of BlaC D233A and D246A were performed. All simulations were performed for both protonation states of the non‐mutated Asp residue and the data are reported in the supplementary material, but here we focus the discussion on the results of the Asp233‐OH/D246A and D233A‐Asp246‐O^−^ simulations because those protonation states yielded stable interactions in BlaC WT. For the mutants, no crystal structures are available, so the mutation was modeled by removing the side chain atom in the structure of BlaC WT (pdb_00002gdn (Wang et al., 2006)). The distances between the C*β* atoms of the residues in positions 233 and 246 (d_D233C*β*–D246C*β*_) were measured. For WT BlaC, two states are observed, with distances of 0.57 and 0.70 nm. In the latter state, Asp233 assumes a different χ_1_ rotamer and is slightly translated compared to the crystal structure, causing a larger C*β*–C*β* distance, while maintaining the H‐bond with Asp246. In BlaC D233A and D246A, the distance fluctuates gradually between 0.5 and 0.8 nm, in line with the absence of specific states caused by side‐chain interactions due to removal of the H‐bond capability (Figure S3). The effect of the mutations to Ala on the catalytic residues was determined by analyzing correlations between distances between active site residues in the MD simulations represented as a 2D probability histogram. A correlation distance analysis describes how two interatomic distances vary relative to each other over the course of the simulation, revealing whether changes in one distance are associated with changes in the other and, thus, identifying coordinated motions. The correlations between the distances between the catalytic residues Ser70, Lys73, Ser130, Glu166 and K234 (Figure S6) were analyzed. The correlation plots (Figure 3a) show three dominant states: state A (centered at 0.30 nm for Ser70 Oγ–Lys73 Nζ [d_S70–K73_] and 0.39 nm for Ser70 Oγ–Ser130 Oγ [d_S70–S130_]), state A′ (0.30 nm d_S70–K73_; 0.42 nm d_S70–S130_) and state B (0.85 nm d_S70–K73_; 0.81 nm d_S70–S130_). State A is the dominant one in WT BlaC. In both variants a change to state A′ is observed and in BlaC D246A also state B becomes more populated. The difference between state A and state A′ is a larger d_S70–S130_ in the latter. For each state, the structure closest to the mean of the state area defined by probabilities higher than 1.2 × 10^−3^ was extracted as the representative structure (Table S3). The structure of state A of BlaC WT and the crystal structure are quite similar (RMSD = 0.125 nm, Figure S7). States A′ of BlaC D233A and BlaC D246A show changes in the conformations of Ser130 and Lys234. The H‐bonds of Ser130 with Ser70 and Lys234 are lost and a new one is formed between Lys234 and Thr216, correlated with an alternative orientation of Ser130 (Figure 3b). Eliminating the interaction between Asp233 and Asp246 could affect the interaction between Asn214 and Asp233 (Figure 1b), which could promote an alternative orientation of the loop 213–220 and change the interaction between Lys234 and Thr216. Therefore, we propose that the interaction between Asp233 and Asp246 helps maintain the active site conformation via the interactions with loop 213–220. The orientation of the Ser130 sidechain appears critical for hydrogen bond formation to Ser70. BlaC crystal structures show a χ_1_ dihedral angle for Ser130 between 75° and 90°, compatible with a short distance between the Oγ atoms of Ser130 and Ser70 and therefore assumed to be the catalytically most active state. However, in some structures (PDB: 7A74, 7A5W, 8R88) the sidechain is oriented away from the active site (χ_1_ = 160°–180°), suggesting that it can access this alternative orientation. Surprisingly, in the BlaC WT simulations, Ser130 explores angles between 60° and 90° with a probability of only 33% of the sampled conformations. The highest populated state (67%) has the sidechain of Ser130 rotated away (χ_1_≈170°). In BlaC D233A and D246A, this balance is even more skewed. The mutants explore the angle range between 75° and 90° only 3.9% and 4.7% of the time, respectively (Figures 3c and S8), which is statistically very significantly different from BlaC WT.

### 2.5 BlaC D246A shows an alternative conformation of Ser70, disrupting the interaction between Asn245 and Phe68

**FIGURE 4:**
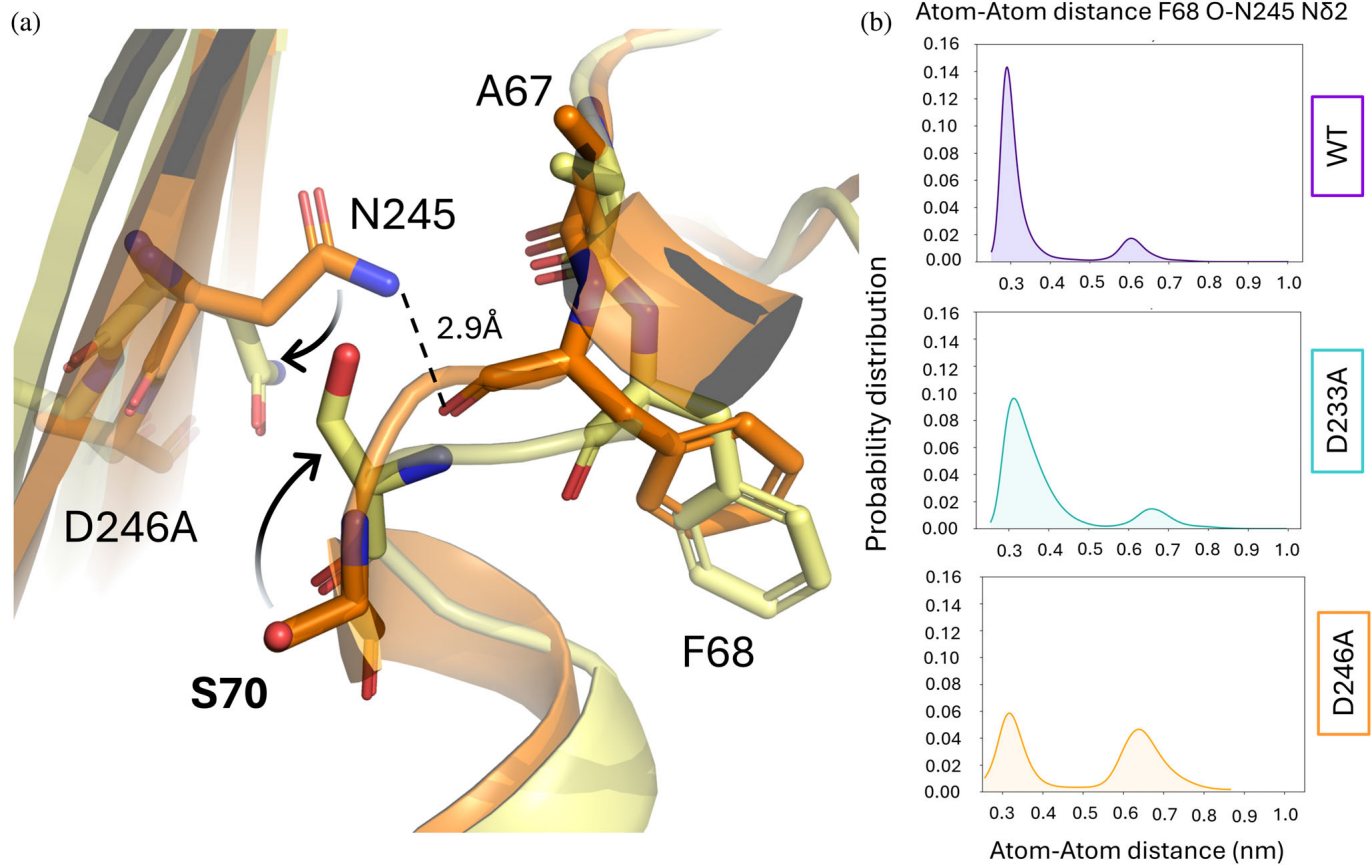
Conformational change in BlaC D246A as observed in MD simulations. (a) Residues Ala67, Phe68, Ser70, Asn245, and Ala246 are shown as sticks, and the catalytic residue is indicated with a label in bold. The superimposed structures correspond to BlaC D246A states A′ (orange) and B (pale yellow) (extracted from replicate 2 at 867 ns). The arrows indicate conformational changes in some residues, and the H‐bond between Asn245 and Phe68 is shown as a dashed line. (b) Probability distribution of the distance between the Phe68 O and the Asn245 Nδ2 atoms for BlaC WT, D233A, and D246A. Probability distributions were computed from 5 × 1 μs of MD simulations. Kolmogorov–Smirnov tests show that the mutant distributions differ significantly from the WT distribution, yielding *D* = 0.316 for D233A and *D* = 0.476 for D246A (both *p*<< 0.001).

For BlaC D246A, also state B is notably more populated (Figure 3a). A representative structure displays an alternative conformation of residue Asn245, disrupting the H‐bond between Phe68 O and Asn245 Nδ2 (d_F68–N245_, Figure 4a), leading to an increased distance between loop 61–68 and *β*‐strand 4, thereby affecting the position of residue Ser70. In the simulations, d_F68–N245_ mostly remains between 0.3 and 0.4 nm for BlaC WT and D233A. This conformation is relatively stable, showing only 35 (WT) and 16 (D233A) transitions between the 0.25–0.45 nm and 0.6–0.8 nm distance ranges and exhibiting an average residence time of 604 ns (WT) and 412 ns (D233A) (Figure S3). In simulations of BlaC D246A, the interaction is disrupted more frequently, showing 165 transitions and 156 ns as the average residence time and state B is more populated (Figure 4b). This state appears incompatible with catalysis, which may provide an explanation for the more detrimental effect of the mutation D246A. Equivalent conclusions can be drawn from the Asp233‐O^−^/Asp246‐OH correlation distance analysis of active‐site residues. D233A maintains a conformational equilibrium comparable to WT, whereas D246A samples a broader range of conformations over the simulation timescale (Figure S6).

## 3 DISCUSSION

Second‐shell residues are thought to play a critical role in ensuring the precise positioning of the active site residues. Here, we examined the interaction between second‐shell Asp233 and Asp246 using the variants D233A and D246A in BlaC. Both Asp233 and Asp246 each exhibit 96% conservation across class A *β*‐lactamases (Chikunova & Ubbink, 2022). In some *β*‐lactamases, such as TEM‐1 and SHV, position 246 is occupied by an isoleucine and an Asp is found at position 214. In these cases, Asp233 interacts with Asp214, thus substituting for the interaction between Asp233 and Asp246. In TEM‐1, this interaction has been shown through simulations to be essential for maintaining active‐site integrity; in its absence, the active site becomes destabilized (Roccatano et al., 2005). Asp246 was highlighted in QM/MM calculations for its modulatory role in the electrostatics for carbapenem activity (Jabeen et al., 2024) and in flexibility modulation in the context of evolutionary pathways (Modi et al., 2021). The SHB between Asp233 and Asp246 was characterized for CTX‐M by Kemp et al. (Kemp, Nichols, et al., 2021) Using variant D233N, the SHB was replaced by an ordinary HB, resulting in decreased activity for various substrates and reduced affinity for inhibitors. B‐factor analysis of the crystal structure indicated increased flexibility in the 213–219 loop (Kemp, Nichols, et al., 2021). Earlier work on BlaC showed that replacement of both Asp233 and Asp246 with Asn yielded stable and active protein. Mutations to Ala affected stability and activity more, but active protein was still present. The mutations of Asp246 had more effect than those of Asp233 (Chikunova & Ubbink, 2022). We wanted to establish the importance of the H‐bond between the two Asp residues, so we decided to eliminate the H‐bond entirely and mutated the residues to Ala. For most substrates, these mutations decrease catalytic efficiency compared to WT, with BlaC D246A consistently showing a more pronounced effect. Both variants are equally less thermostable than WT BlaC under equilibrium unfolding conditions. When thermostability is measured under non‐equilibrium conditions, BlaC D233A and D246A unfold less cooperatively than WT BlaC, and again this effect is more pronounced in the D246A variant, typical of proteins that are less tightly packed (Magliery, 2015; Munson et al., 1996). Surface mutations tend to be less destabilizing than mutations in the core of the protein (Faure & Koonin, 2015; Nisthal et al., 2019; Schwersensky et al., 2020), so the fact that Asp246 is more buried than Asp233 may offer an explanation for its reduced functional performance upon mutation to Ala. The 2D‐NMR analysis revealed subtle structural rearrangements distributed across a large part of the protein, including the active site region, in line with roles as second‐shell residues. It is noted that amide chemical shifts are very sensitive to changes in the hydrogen bond network, including the water layer, so CSPs do not necessarily represent significant changes in atom positions. Also, changes in affinity for solvent ions can cause CSP. BlaC is known to bind a phosphate in the active site region (Elings et al., 2017). We attempted to detect the hydrogens in the SHBs with NMR spectroscopy. Due to the large deshielding, they are expected to resonate downfield of the envelope of ^1^H signals (Langkilde et al., 2008). However, even at low temperature (−5°C), we did not detect the signals, probably due to rapid exchange with the hydrogens in the bulk water. MD simulations were used to visualize the structural effects of the mutations. LBHBs cannot be accurately simulated with regular MD simulations with fixed electrostatics, because in this approach the hydrogen is attached to one of the two Asp residues rather than shared. For this reason, we determined whether the H‐bond between Asp233 and Asp246 would be stable in these simulations. Protonation of Asp233 and deprotonation of Asp246 yielded satisfactory results. The structure of WT protein remains very similar to that observed in the crystal structure over the duration of the simulations (state A). A surprising finding is that Ser130 is rotated away from Ser70 for a large part of the time, although this rotamer has been observed also in several crystal structures. For the variants BlaC D233A and D246A this rotamer is even more populated and a state A′ is explored, in which also Lys73 is in a different orientation (Figure 3b). In BlaC D246A an additional conformation (state B) is more populated, in which the HBs between the sidechain of Asn245 and backbone atoms of Ala67 and Phe68 are lost, causing a reorientation of the 61–68 loop (Figure 4a). As in both mutants the H‐bond between the two Asp residues is abolished, the differences observed for BlaC D233A and D246A must be attributable to other effects, such as difference in packing of the two residues.

It is concluded that, in general, the simulations indicate that abolishing the interaction between Asp233 and Asp246 leads to subtle shifts in populations of accessible conformational states (A, A′, and B) of the active site and its surroundings, in line with a role for active site tuning as second‐shell residues. Such changes align with the experimental results of changes in activity and widespread CSPs. However, it is also important to consider the limitations of MD. The fixed electrostatics cannot account for (de)protonations and the delocalised nature of a LBHB cannot be taken into account. The simulations were run on the resting state of BlaC and conformational populations may shift upon interaction with the substrate. Furthermore, the catalysis timescale is milliseconds, whereas simulations reach the low microsecond range, and it cannot be excluded that other conformations have low free energy but can only be reached on a longer timescale due to high transition barriers. Thus, these results cannot be directly linked to catalytic properties, although it seems unlikely that a conformation of Ser70 as observed in state B would be compatible with catalysis. This state is more populated in BlaC D246A, which is also the mutant most affected in activity. The surprising finding of the χ_1_ distribution of Ser130 warrants further investigation. In the classical mechanistic model, the role of Ser130 is to accommodate hydrogen transfer during catalysis and this seems incompatible with a χ_1_ close to 170°.

## 4 CONCLUSION

Mutations D233A and D246A modify the non‐covalent interaction network in BlaC but still yield a functional protein, despite disrupting a highly conserved interaction. Within the limitations of the technique, the MD simulations provide models for the changes in conformations and dynamics that may help to interpret the experimental effects on activity and stability in structural terms. We conclude that the elimination of this interaction leads to a subtle shift in the conformational equilibria of catalytic residues, in line with a role tuning the active site of the enzyme.

## 5 MATERIALS AND METHODS

### 5.1 Mutagenesis

Site‐directed mutagenesis was performed using whole plasmid site‐directed mutagenesis with a pET28a plasmid carrying the *blaC* gene lacking codons for the N‐terminal 42 amino acids that constitute the signal peptide and using the genetic code for a TEV cleavable N‐terminal His(6)‐tag (Figure S10). Primer sequences are given in Table S4. The WHOPS product was incubated at 37°C for 60 min with *Dpn*I to remove the template plasmid, followed by 30 min at 72°C with *Pfu* DNA polymerase, and an additional 60 min at 37°C with *Dpn*I. Afterward, super‐competent *E. coli* DH5*α* cells (Inoue et al., 1990) were transformed with the WHOPS product, plated on LB agar with 50 μL/mL of kanamycin, and incubated overnight at 37°C. Single colonies were picked to inoculate LB cultures, which were incubated overnight at 37°C. Plasmid was isolated using a GeneJET Plasmid Miniprep Kit (Thermo Fisher Scientific). Purified DNA was amplified via standard PCR. Amplified DNA was further purified using a GFX PCR DNA Purification Kit (Illustra), and the presence of the mutation was confirmed by Sanger sequencing.

### 5.2 Protein production and purification

The pET28a plasmids encoding the *blaC* WT, D233A, and D246A genes were expressed in *E. coli* BL21(DE3) pLysS. The proteins were produced and purified as described (Elings et al., 2017). Pure protein was stored in 100 mM sodium phosphate buffer at pH 6.4 by flash freezing in liquid nitrogen and kept at −70°C.

### 5.3 Thermal shift assay

The thermal stability was assessed by two methods, using 10 μM protein in sodium phosphate buffer (100 mM) at pH 6.4. Changes in the tryptophan fluorescence ratio at 350 and 330 nm were monitored using a Tycho NT.6 (NanoTemper Technologies) across a temperature range of 35 to 95°C in triplicate to calculate the inflection temperature (*T*_i_). The melting point (*T*_m_) was obtained by a thermal shift assay with SYPRO Orange dye (Invitrogen). Data were acquired using the CFX 96 Touch Real‐Time PCR Detection System (Bio‐Rad) over a temperature range of 20–100°C, with 1°C/min increments, in triplicate. The *T*_i_ and *T*_m_ were calculated from the derivatives of the curves, and the error was determined from the standard deviation of the measurements in triplicate.

### 5.4 Enzyme kinetics

The kinetic experiments were performed in triplicate in 100 mM sodium phosphate buffer, pH 6.4, at 25°C. Initial velocities were calculated by measuring the slope of the first 120 s of each curve. Three measurements were averaged, and the standard deviation was used as the error. All data were collected on a PerkinElmer Lambda 1050+ UV–vis spectrometer fitted with a thermostat. For hydrolysis of ampicillin and penicillin G, the change in absorbance at 235 nm was monitored for 5 min using 2 nM BlaC and 100 μM substrate. For nitrocefin, the absorbance change at 486 nm was measured using 100 μM substrate and 5 nM enzyme over 3 min. For cefuroxime, cefoxitin, ceftriaxone, and cefepime, the absorbance change at 230 nm was recorded with 25 μM substrate and 1 μM enzyme over 3 min. Data were analyzed using Python (v3.10). Data handling and preprocessing were performed with pandas (v1.5) and NumPy (v1.23). Data visualization was performed with Matplotlib (v3.7). Michaelis–Menten curves were fitted by nonlinear least‐squares regression using the curve_fit function in SciPy (v1.10) according to Equation (1).

(1) $$v_{0}=\frac{k_{\mathrm{cat}}\left[E\right]\left[S\right]}{K_{M}^{\mathrm{app}}+\left[S\right]}$$

where *v*_0_ is the initial reaction velocity, *k*_cat_ is the turnover number, [*S*] is the initial substrate concentration, [*E*] is the total enzyme concentration, and *K*_M_^app^ is the apparent Michaelis–Menten constant (Sun et al., 2024).

The following extinction coefficients were used. Nitrocefin: Δ*ε*_486_ = 17.776 mM^−1^ cm^−1^, ampicillin: Δε_235_ = 0.861 mM^−1^ cm^−1^, penicillin G: Δε_235_ = 0.861 mM^−1^ cm^−1^ (personal communication Saar Koene), ceftazidime: Δε_260_ = 7 (1) mM^−1^ cm^−1^, cefepime: Δε_260_ = 10.4 (0.8) mM^−1^ cm^−1^, cefoxitin: Δε_260_ = 8.3 (0.4) mM^−1^ cm^−1^, cefuroxime: Δε_260_ = 8.3 (0.5) mM^−1^ cm^−1^, and ceftriaxone: Δε_260_ = 7.8 (0.4) mM^−1^ cm^−1^ (Sun et al., 2024).

### 5.5 NMR spectroscopy experiments

^1^H–^15^N TROSY‐HSQC and HNCA spectra were recorded on a Bruker AVIII HD 850 MHz spectrometer at 25°C on 0.5 mM (^15^N, ^13^C) BlaC D233A and D246D in 100 mM sodium phosphate buffer at pH 6.4 with 6% D_2_O in a 3‐mm NMR tube. Data were processed in Topspin 4.1.4 (Bruker), and spectra were analyzed with CCPNmr Analysis V3 software (Skinner et al., 2016). The recorded WT spectra were compared to BMRB entry 27,067 (Elings et al., 2017), which reports the chemical shifts for 98.5% of the backbone amides. The peaks in the spectra of BlaC D233A and D246A were assigned by comparison to those in the WT BlaC spectrum and checked using the HNCA spectra. The assignments of the variants have been deposited in the BMRB under entries 53,973 (D233A) and 53,974 (D246A). Average CSPs relative WT BlaC, Δ*δ*, of the ^1^H (Δ*ω*_1_) and ^15^N (Δ*ω*_2_) resonances of backbone amides were calculated using Equation (2).

(2) $$\Delta \;\delta =\sqrt{\frac{1}{2}\left(\Delta \;\omega_{1}^{2}+{\left(\frac{\Delta \;\omega_{2}}{5}\right)}^{2}\right)}$$

### 5.6 Molecular dynamics simulations

The initial structure for WT BlaC was pdb_00002gdn (Wang et al., 2006). To obtain the initial structures for BlaC Asp233 or Asp246, the WT structure was mutated by erasing all the atoms from the side chain except for the *β*‐carbon. Hydrogen atoms were added in GROMACS, and the system was parameterized using the AMBER99SB‐ILDN force field for proteins (Lindorff‐Larsen et al., 2010). Protonation states of ionizable residues were assigned according to their predicted p*K*_a_ values at pH 7.0, except for Asp179, which was selected to be in a protonated form and in the pair Asp233/Asp246 one of the two was protonated. The protein was placed in a dodecahedron water box extending at least 1.2 nm beyond any protein atom and solvated with TIP3P water molecules (Jorgensen et al., 1983). Sodium and chloride ions were added by replacing random water molecules at 100 mM, ensuring overall charge neutrality. Five replicates of 1 μs MD simulations were performed, starting from the same atomic positions, and with different velocities, randomly assigned from the Maxwell–Boltzmann distribution at 25°C for each run. Simulations were performed using the force field mentioned above. Energy minimization was performed using the conjugate gradient algorithm until the maximum force on any atom was below 150 kJ mol^−1^ nm^−1^. The system was equilibrated in two stages with harmonic positional restraints in three directions with a force constant of 1000 kJ mol^−1^ nm^−2^ on the protein, first under constant number of particles, volume, and temperature (NVT ensemble) conditions, followed by equilibration under constant number of particles, pressure, and temperature (NPT ensemble). MD were performed using a 2 fs time step for 1 μs, a velocity‐rescaling thermostat (Bussi et al., 2007) at 25°C, and a Parrinello–Rahman barostat (Parrinello & Rahman, 1981) at 1.0 bar. Long‐range electrostatics were treated with the particle‐mesh Ewald method, and short‐range cutoffs of 1.1 nm were used for both electrostatics and van der Waals interactions. Bonds involving hydrogen were constrained using LINCS (Hess et al., 1997).

Prior to analysis, solvent molecules were removed from the trajectories. The initial 50 ns of each trajectory were discarded to ensure that only equilibrated portions of the simulations were included in the analyses. The structures remain stable during the trajectories, as is evident from the Cα‐RMSD values that stabilized at 0.15, 0.13, and 0.12 nm for BlaC WT, D233A, and D246A (protonation state Asp233‐OH/Asp246‐O^−^) and at 0.13, 0.14, and 0.12 nm for BlaC WT, D233A, and D246A (Asp233‐O^−^/Asp246‐OH), respectively. Interaction correlations were determined by plotting the distance distribution of one interaction against that of the other in a 2D plot using a bin width of 0.007 nm. The probability histograms were then smoothed using a kernel density function with a bandwidth of 2. These calculations were performed and plotted in Python 3.11.5 together with the following libraries: NumPy 1.26.2 (Harris et al., 2020), pandas 2.1.3 (McKinney, 2010), MDTraj 1.9.9 (McGibbon et al., 2015), Seaborn 0.13.2 (Waskom, 2021), and Matplotlib 3.8.0 (Hunter, 2007). To visualize the conformation of high‐probability density areas (>0.012), trajectories were filtered to retain structures with both distances within a given range (Table S3). The trajectory frame closest to the Cα‐RMSD mean of the filtered structures was taken as the representative conformation of that state. The χ_1_ dihedral angle of Ser130 was determined each ns of the whole trajectory and the probability distribution was calculated with a bin size of 10°. The average residence time was calculated for the d_F68–N245_ window from 0.25 to 0.45 with a minimum residence time of 1 ns and a maximum allowed gap of 1 ns. The Kolmogorov–Smirnov test is a nonparametric test that compares the normalized cumulative distribution functions of two samples to address whether they are significantly different, here this test is used to compare probability distributions of χ_1_ dihedral angle of Ser130 and atom‐atom distances. *D* is the test statistic, defined as the maximum absolute distance between the two distributions (ranging between 0 and 1), and *p* is the corresponding *p*‐value, obtained via the Kolmogorov distribution. Statistical significance is strongly dependent on the sample sizes of the two distributions, which are very high (*n* = *m* = 4750) when considering every ns in the MD simulation, yielding highly defined distributions, see Figure S9 as an example.

## AUTHOR CONTRIBUTIONS

**Cristina V. Lopez‐Gallego:** Writing – original draft; writing – review and editing; validation; methodology; investigation; formal analysis; data curation; conceptualization. **Monika Timmer:** Investigation. **Jocelyne Vreede:** Writing – review and editing; supervision; formal analysis. **Marcellus Ubbink:** Writing – review and editing; supervision; project administration; methodology; funding acquisition; formal analysis; data curation; conceptualization.

## CONFLICT OF INTEREST STATEMENT

The authors declare that they have no known competing financial interests or personal relationships that could have appeared to influence the work reported in this paper.

## Supporting information


**Figure S1.** Michelis–Menten plots of BlaC WT (black), D233A (turquoise) and D246A (orange) for hydrolysis of ampicillin, nitrocefin, cefuroxime and ceftriaxone. The line represents the non‐linear fit to the experimental data points. The error bars represent the standard deviation of the measurement in triplicate.
**Figure S2.** ^1^H–^15^N TROSY‐HSQC spectra of BlaC in sodium phosphate buffer, pH 6.4 at 25°C. (A) Overlay of BlaC D233A (turquoise) and WT (black). (B) Overlay of BlaC D246A (orange) and WT (black).
**Figure S3.** MD time traces of atom‐to‐atom distances. The different colors represent five replicates.
**Figure S4.** MD time traces of the distance between Asp233Oδ1 and Asp246Oδ1 in purple, Asp233Oδ1 and Asp246Oδ2 in turquoise, Asp233Oδ2 and Asp246Oδ1 in orange and Asp233Oδ2 and Asp246Oδ2 in pistachio during BlaC WT with Asp233 (A) or Asp246(B) protonated in the simulation, each of the subplots represents each of the 5 replicates of 1 μs runs.
**Figure S5.** Structural superposition of the x‐ray crystal structure 2GDN, colored in white, and BlaC WT Asp233‐OH/Asp246‐O^−^ d_D233Cγ‐D246Cγ_ state 1 derived from molecular dynamics simulations in slate, BlaC WT Asp233‐O^−^/Asp246‐OH d_D233Cγ‐D246Cγ_ state 1 derived from molecular dynamics simulations in turquoise and BlaC WT Asp233 O^−^/Asp246‐OH d_D233Cγ‐D246Cγ_ state 2 derived from molecular dynamics simulations in pistachio. Active side residues are labeled in bold.
**Figure S6.** Correlation plots of the distances between indicated active site atoms in five replicates of 1 μs MD simulations of BlaC WT, D233A, and D246A in different protonation states. The color of the areas indicates the probability density.
**Figure S7**. Structural superposition of the x‐ray crystal structure 2GDN, colored in white, and BlaC WT state A derived from molecular dynamics simulations, colored in slate, showing an RMSD of 0.125 nm.
**Figure S8.** Dihedral angle *χ*_1_ time traces of Ser130 in the MD simulations. Different colors represent five different replicates.
**Figure S9.** Kolmogorov–Smirnov (KS) test comparing the cumulative probability distributions of the Ser130 χ1 dihedral angle for WT (indigo), D233A (turquoise), and D246A (orange) across five independent replicas of each system as an illustrative example. KS statistics: WT versus D233A, *D* = 0.2447 (*p* << 0.001); WT versus D246A, *D* = 0.2374 (*p*<< 0.001).
**Figure S10.** The amino acid sequence of BlaC WT used for protein production (Waskom, 2021). Residues corresponding to the N‐terminal 6xHis tag are indicated in cyan, and the TEV protease cleavage site is highlighted in orange. Residues 31–293 correspond to residue numbers 43–307 of the BlaC Uniprot sequence P9WKD3‐1 and are numbered according to Ambler notation. The Gly residues are present at the N‐terminus (residues 29 and 30) to enhance removal of the tag by TEV.

**Table S1.** Kinetic parameters of selected substrates hydrolyzed by BlaC WT, D233A, and D246A at 25°C. The values represent the mean of triplicate measurements, and the errors are the standard deviations. For substrates for which a plateau (Figure S1) was not observed within the detection time, kinetic parameters *k*_cat_ and *K*_m_^app^ could not be independently determined; instead, only the catalytic efficiency (*k*_cat_/*K*_m_^app^) was estimated.
**Table S2.** Distances mean and standard deviations for distances d_D172–D179_ and d_D233–D246_.
**Table S3.** Atoms and distance range used to extract the filtered structures for structural comparison.
**Table S4.** Primer sequences used to introduce mutations into the *blaC* gene.

## Data Availability

The data that support the findings of this study are openly available in Biological Magnetic Resonance Databank at https://bmrb.io/, reference number 53973 and 53974.
