# Supplementary material for: Conserved Asp233–Asp246 hydrogen bond modulates active site dynamics in class A β‐lactamases
Source: Protein Sci. 2026 Sep 17;35(10):e70798. doi: 10.1002/pro.70798 (PMC13584895; doi:10.1002/pro.70798)
Supplement: Supplementary file 1 [file PRO-35-e70798-s001.pdf]

Supplementary figures to

Conserved Asp233-Asp246 Hydrogen Bond Modulates Active Site Dynamics in class A β-lactamases

Cristina V. Lopez-Gallego, Monika Timmer, Jocelyne Vreede, Marcellus Ubbink.

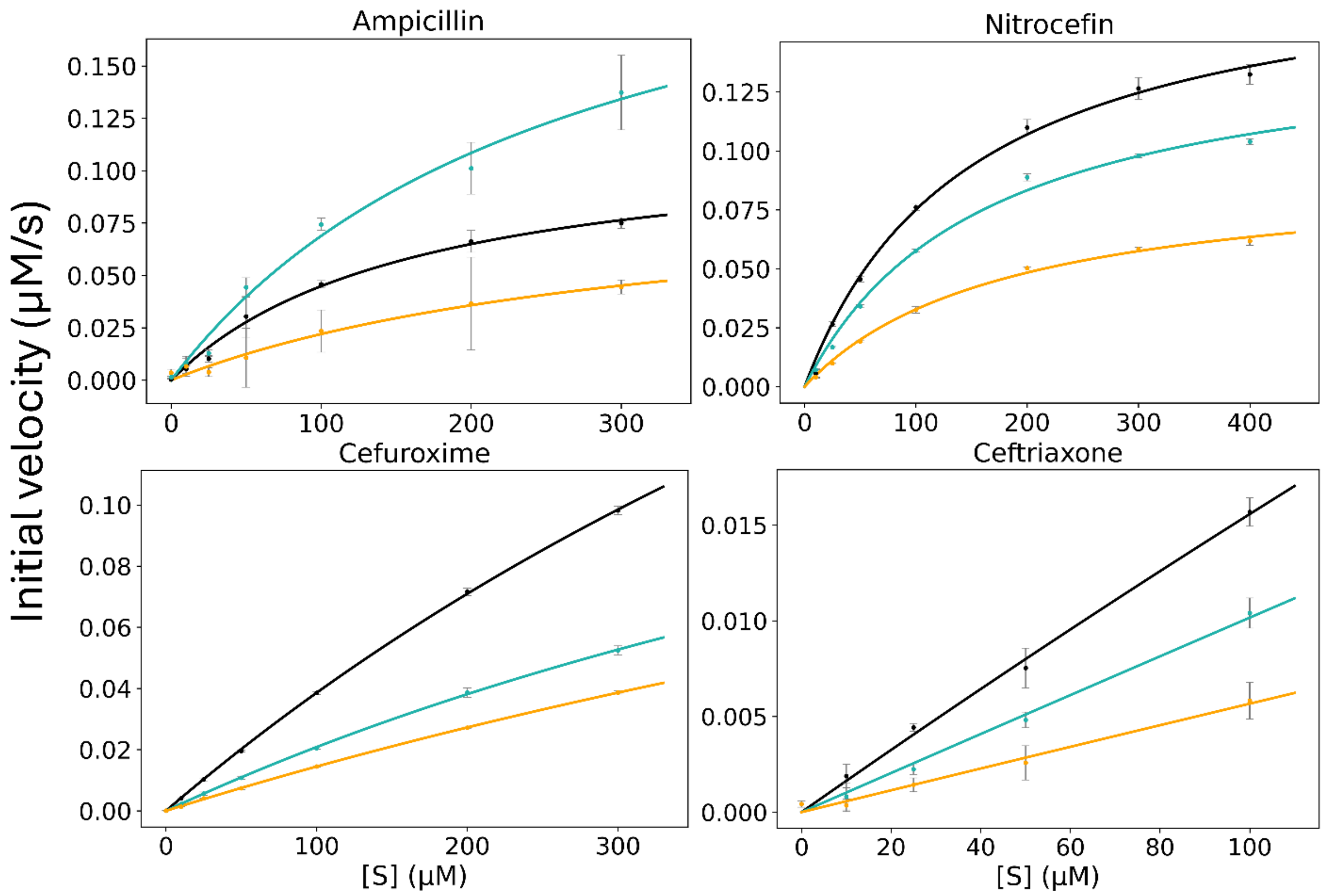

Figure S1. Michelis-Menten plots of BlaC WT (black), D233A (turquoise) and D246A (orange) for hydrolysis of ampicillin, nitrocefin, cefuroxime and ceftriaxone. The line represents the non-linear fit to the experimental data points. The error bars represent the standard deviation of the measurement in triplicate.

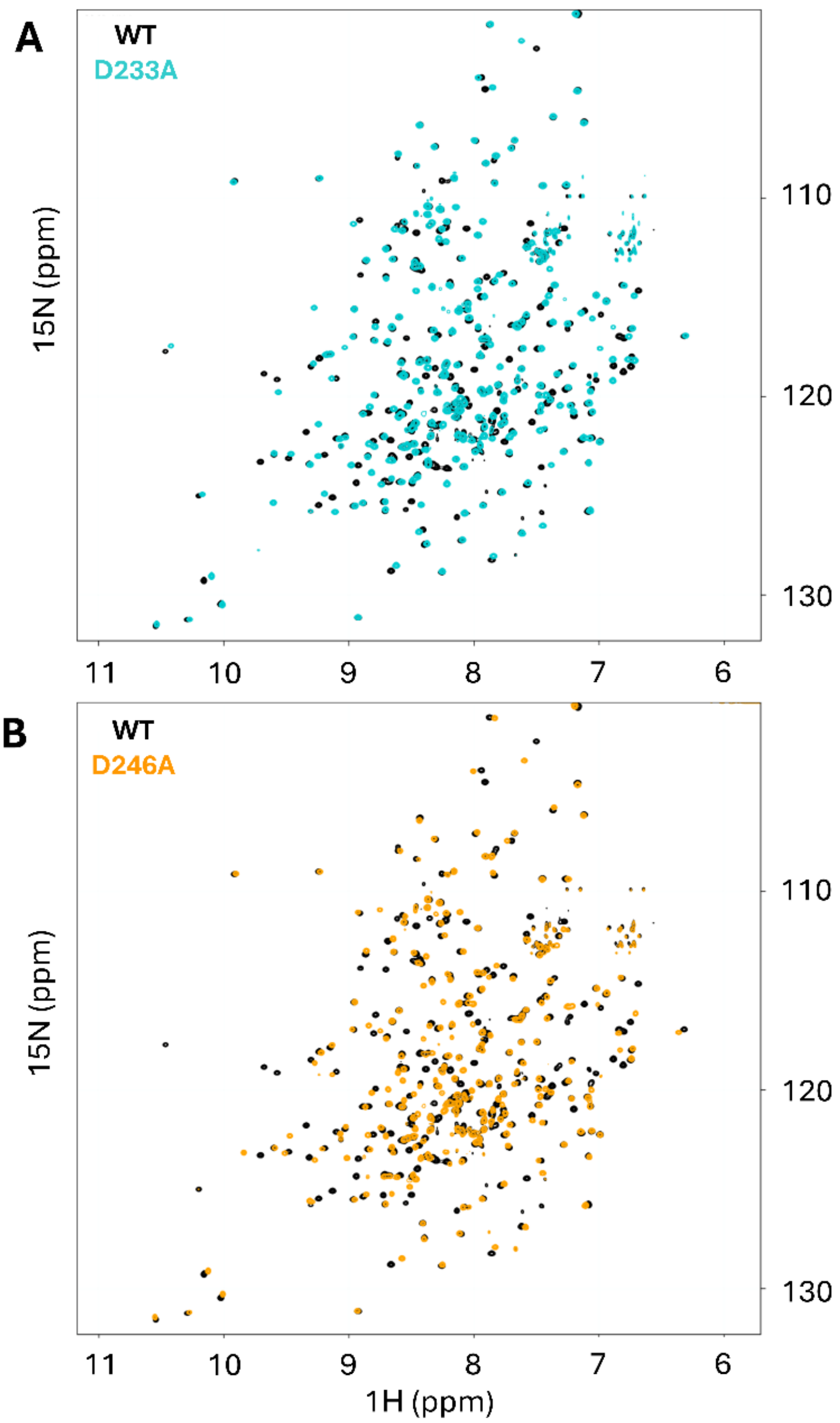

Figure S2. ^1H-^{15}N TROSY-HSQC spectra of BlaC in sodium phosphate buffer, pH 6.4 at 25 °C. A) Overlay of BlaC D233A (turquoise) and WT (black). B) Overlay of BlaC D246A (orange) and WT (black).

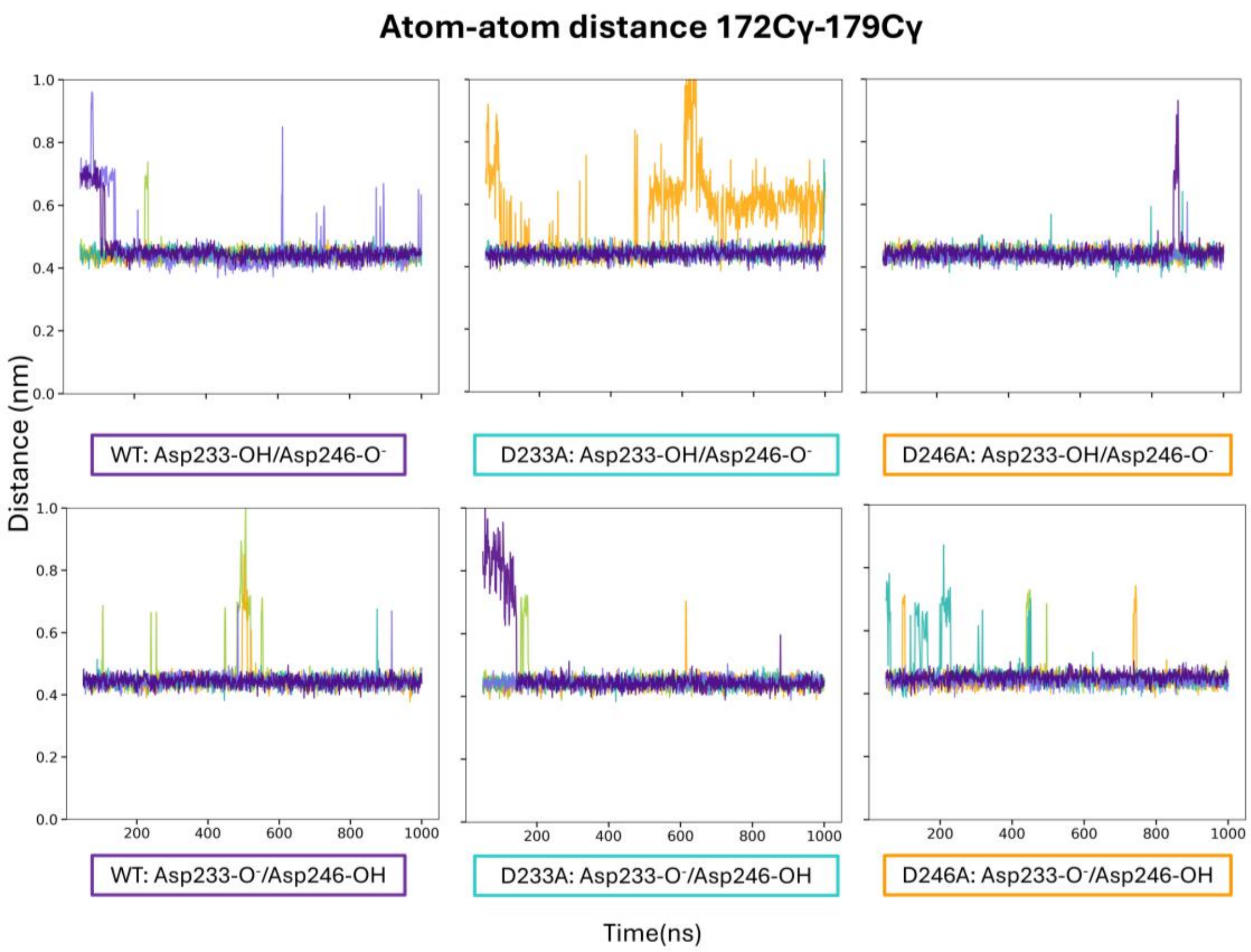
Atom-atom distance 172Cγ-179Cγ
Distance (nm)
Time(ns)
WT: Asp233-OH/Asp246-O-
D233A: Asp233-OH/Asp246-O-
D246A: Asp233-OH/Asp246-O-
WT: Asp233-O-/Asp246-OH
D233A: Asp233-O-/Asp246-OH
D246A: Asp233-O-/Asp246-OH

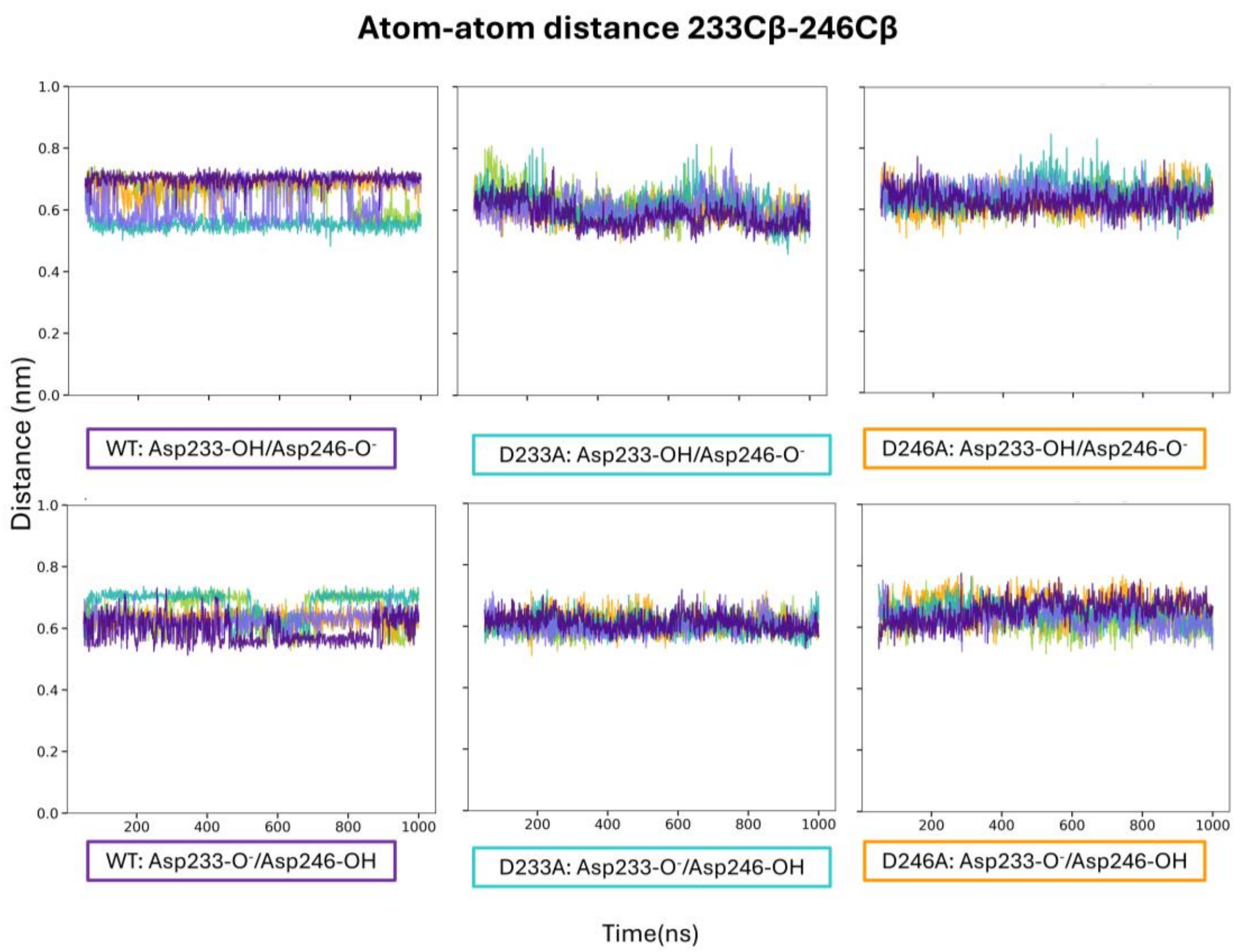
Atom-atom distance 233Cβ-246Cβ
Distance (nm)
Time(ns)
WT: Asp233-OH/Asp246-O-
D233A: Asp233-OH/Asp246-O-
D246A: Asp233-OH/Asp246-O-
WT: Asp233-O-/Asp246-OH
D233A: Asp233-O-/Asp246-OH
D246A: Asp233-O-/Asp246-OH

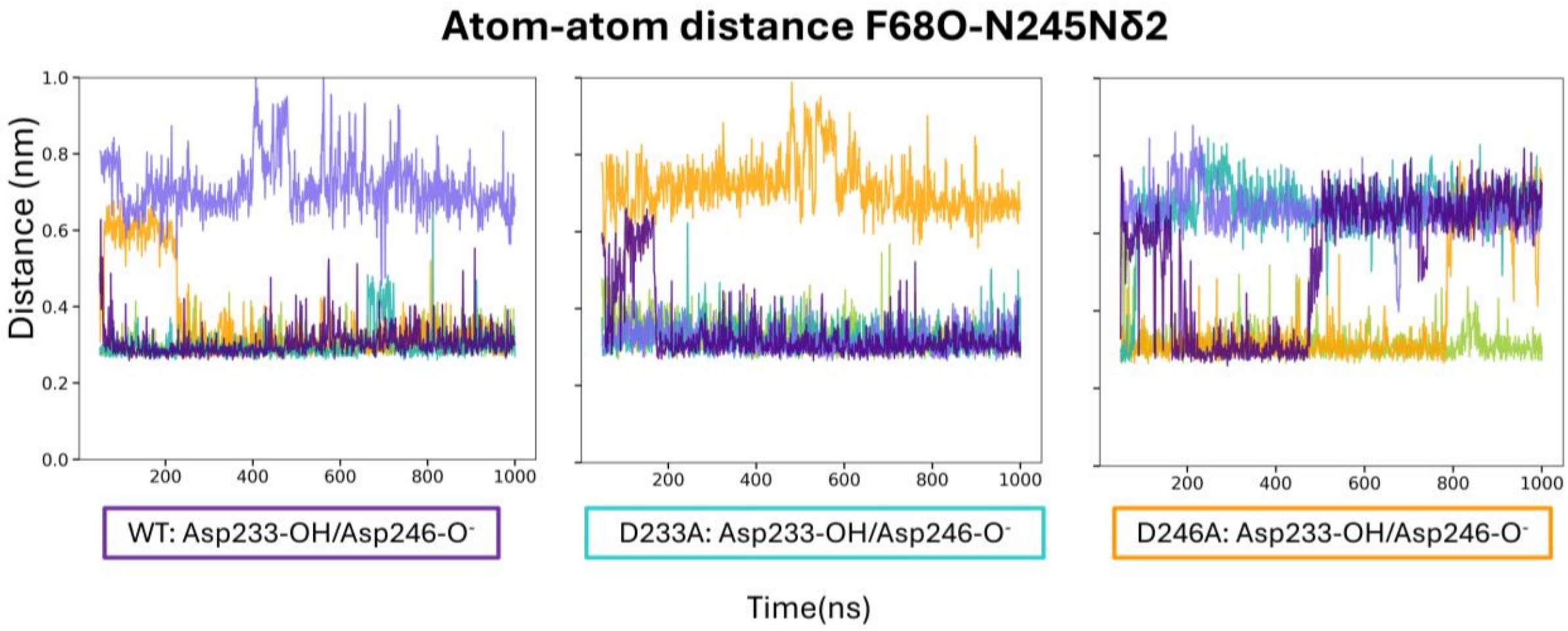

Figure S3. MD time traces of atom-to-atom distances. The different colours represent five replicates.

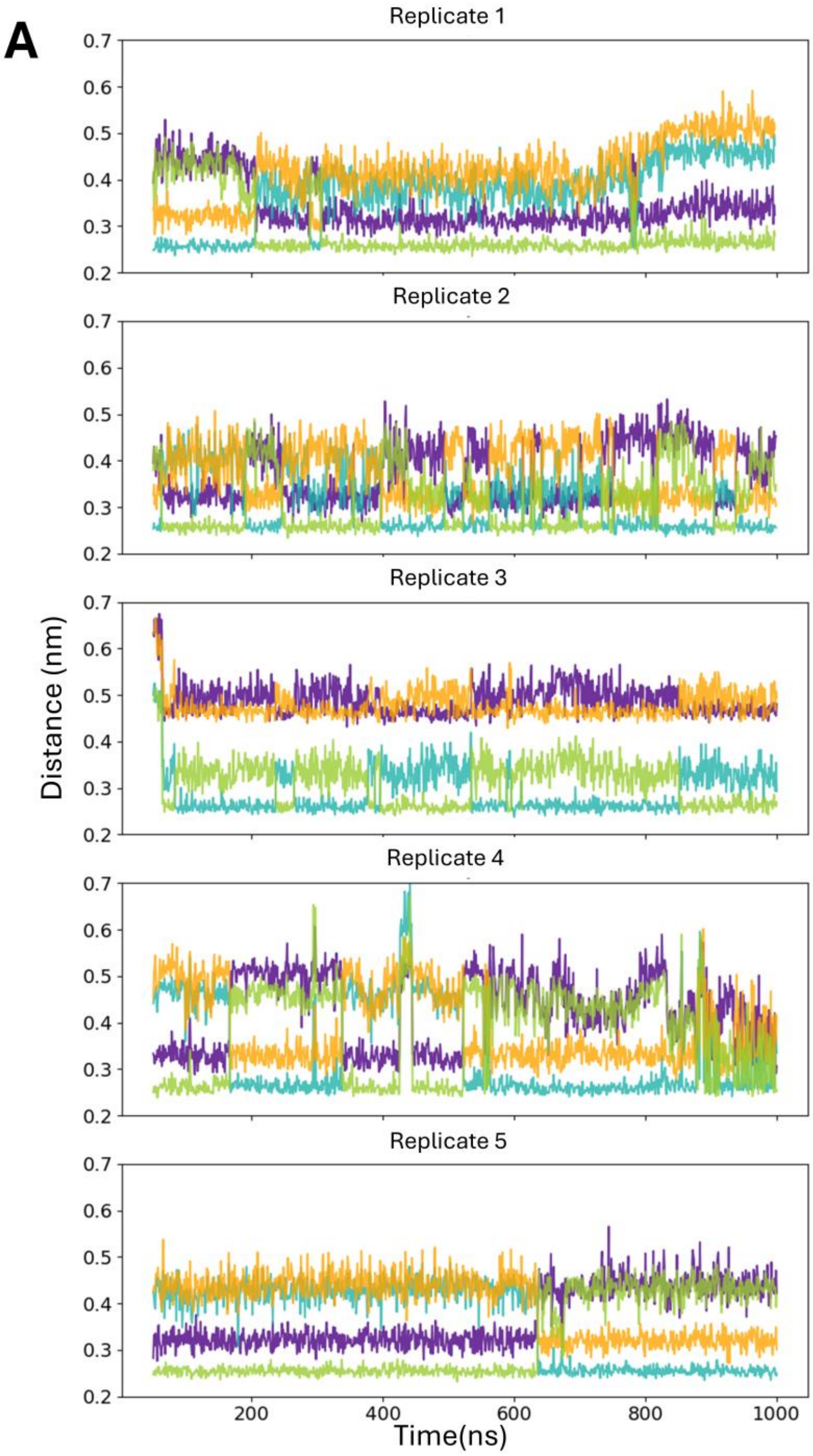
A
Replicate 1
Replicate 2
Replicate 3
Replicate 4
Replicate 5
Distance (nm)
Time(ns)
0.7
0.6
0.5
0.4
0.3
0.2
200
400
600
800
1000

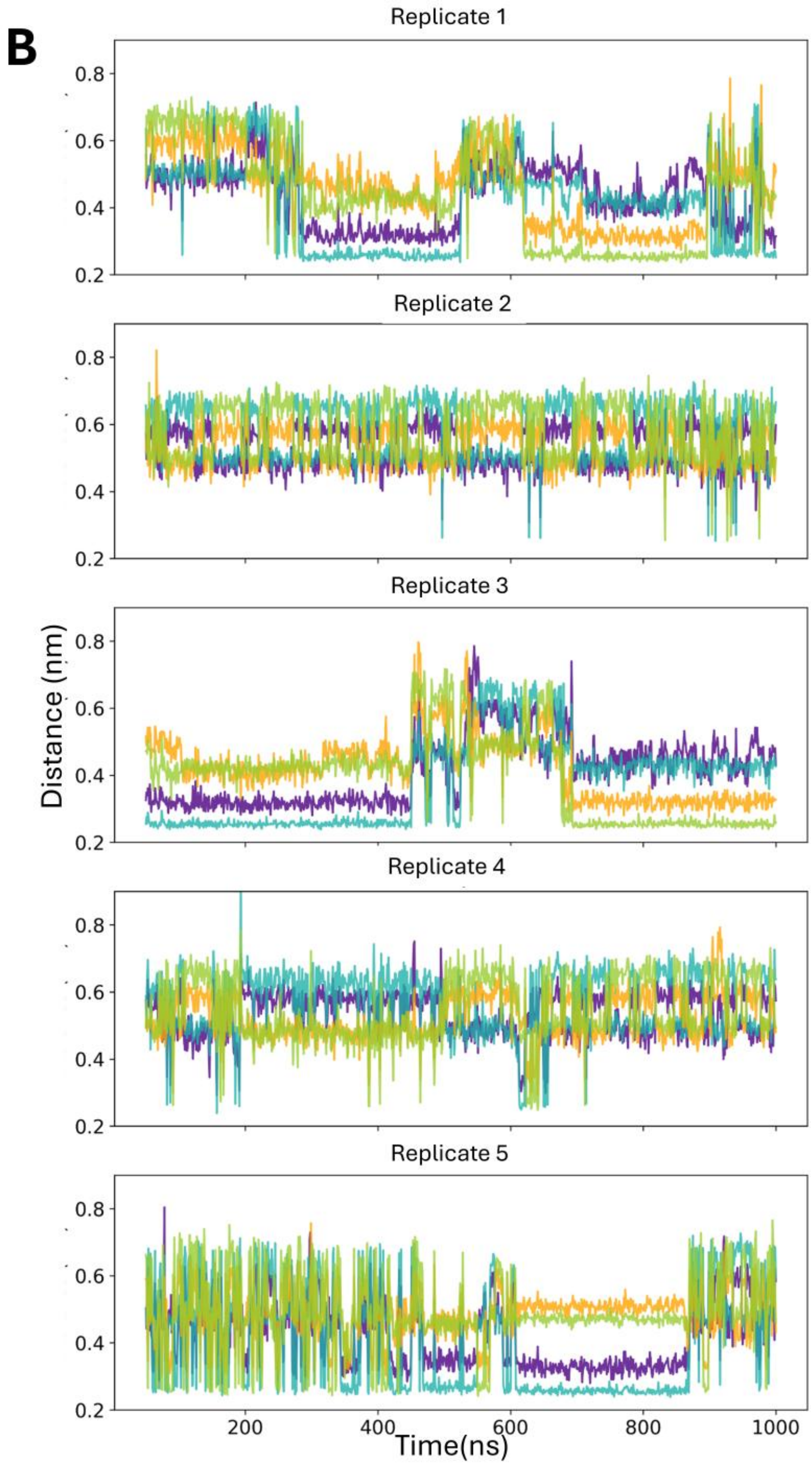

Figure S4. MD time traces of the distance between Asp233Oδ1 and Asp246Oδ1 in purple, Asp233Oδ1 and Asp246Oδ2 in turquoise, Asp233Oδ2 and Asp246Oδ1 in orange and Asp233Oδ2 and Asp246Oδ2 in pistachio during BlaC WT with Asp233 (A) or Asp246(B) protonated in the simulation, each of the subplots represents each of the 5 replicates of 1μs runs.

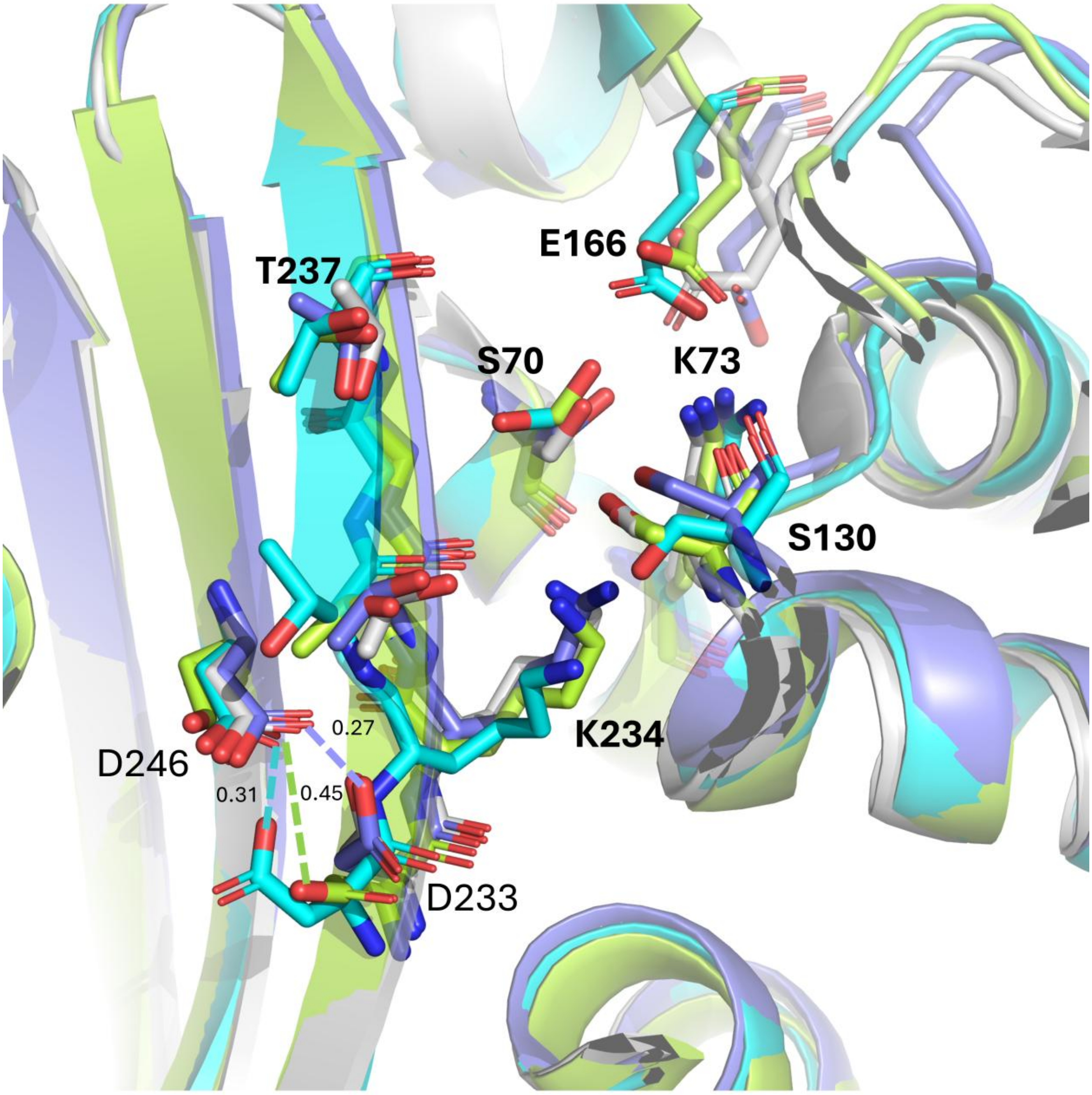

Figure S5. Structural superposition of the X-ray crystal structure pdb_0002gdn, coloured in white, and BlaC WT Asp233-OH/Asp246-O$^-$ $d_{D233C\gamma\text{-}D246C\gamma}$ state 1 derived from molecular dynamics simulations in slate, BlaC WT Asp233-O$^-$/Asp246-OH $d_{D233C\gamma\text{-}D246C\gamma}$ state 1 derived from molecular dynamics simulations in turquoise and BlaC WT Asp233-O$^-$/Asp246-OH $d_{D233C\gamma\text{-}D246C\gamma}$ state 2 derived from molecular dynamics simulations in pistachio. Active side residues are labelled in bold.

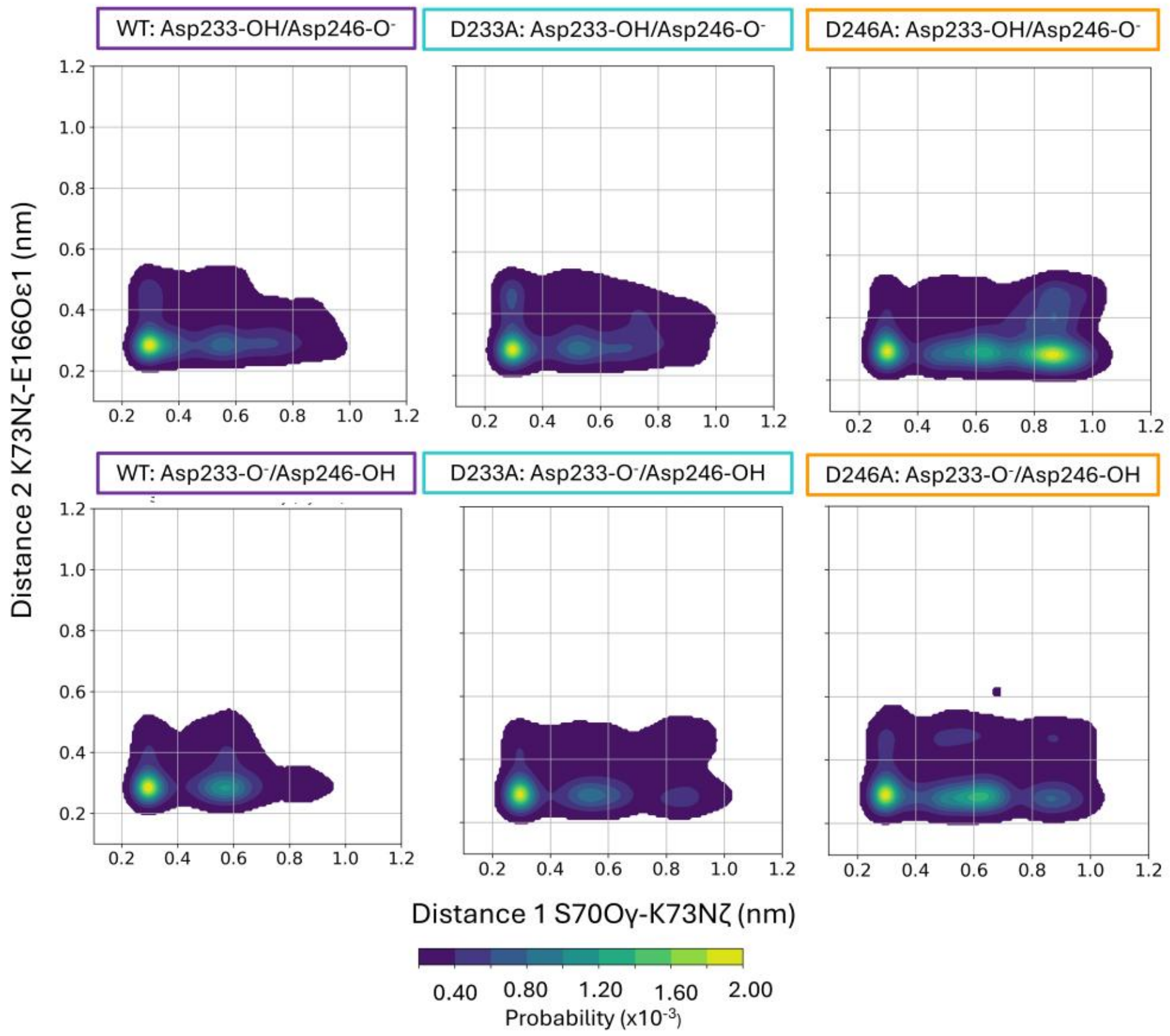
WT: Asp233-OH/Asp246-O-
D233A: Asp233-OH/Asp246-O-
D246A: Asp233-OH/Asp246-O-
WT: Asp233-O-/Asp246-OH
D233A: Asp233-O-/Asp246-OH
D246A: Asp233-O-/Asp246-OH
Distance 2 K73Nζ-E166Oε1 (nm)
Distance 1 S70Oγ-K73Nζ (nm)
0.40 0.80 1.20 1.60 2.00
Probability (x10-3)

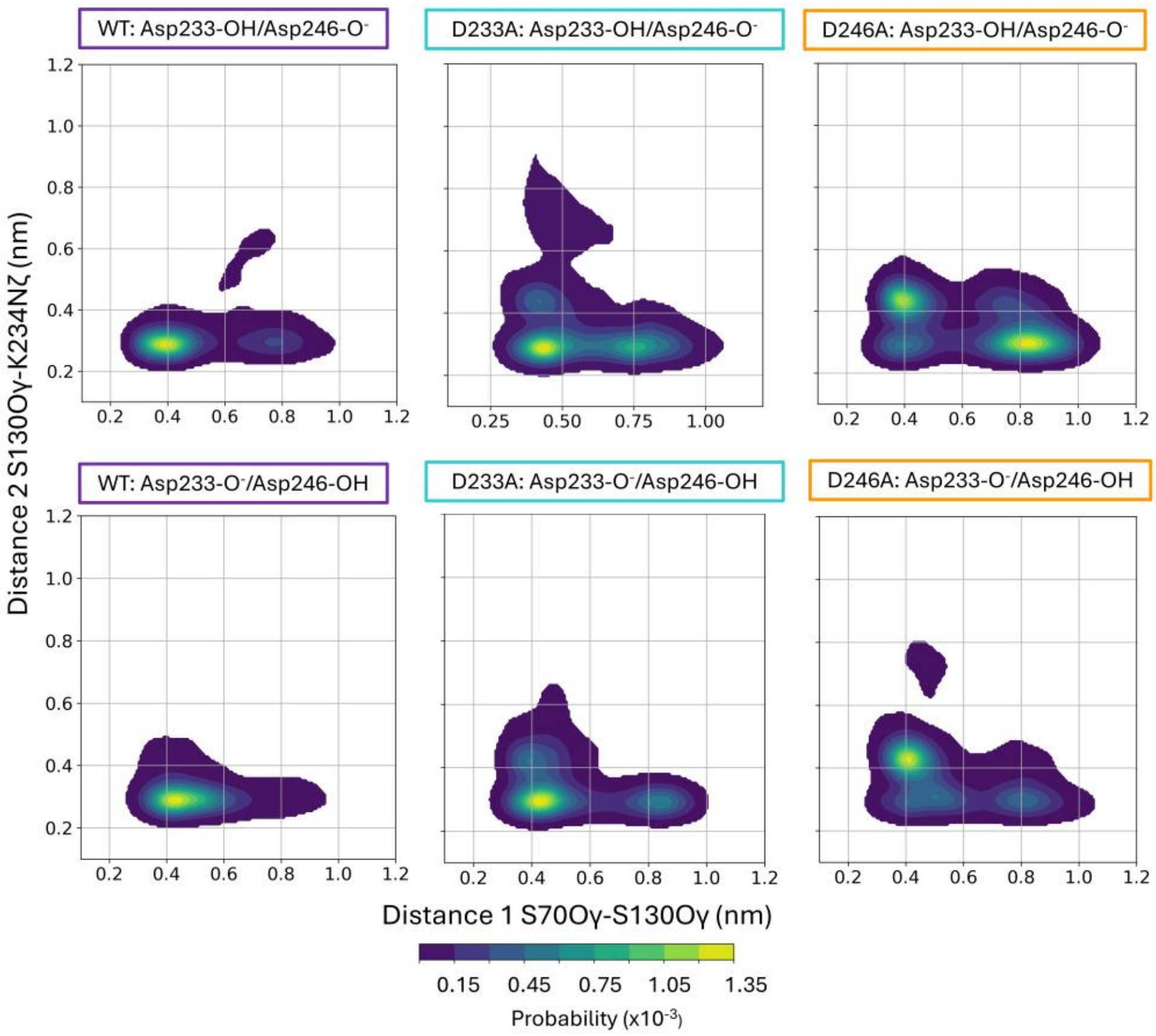
WT: Asp233-OH/Asp246-O-
D233A: Asp233-OH/Asp246-O-
D246A: Asp233-OH/Asp246-O-
WT: Asp233-O-/Asp246-OH
D233A: Asp233-O-/Asp246-OH
D246A: Asp233-O-/Asp246-OH
Distance 2 S130Oγ-K234Nζ (nm)
Distance 1 S70Oγ-S130Oγ (nm)
0.15 0.45 0.75 1.05 1.35
Probability (x10-3)

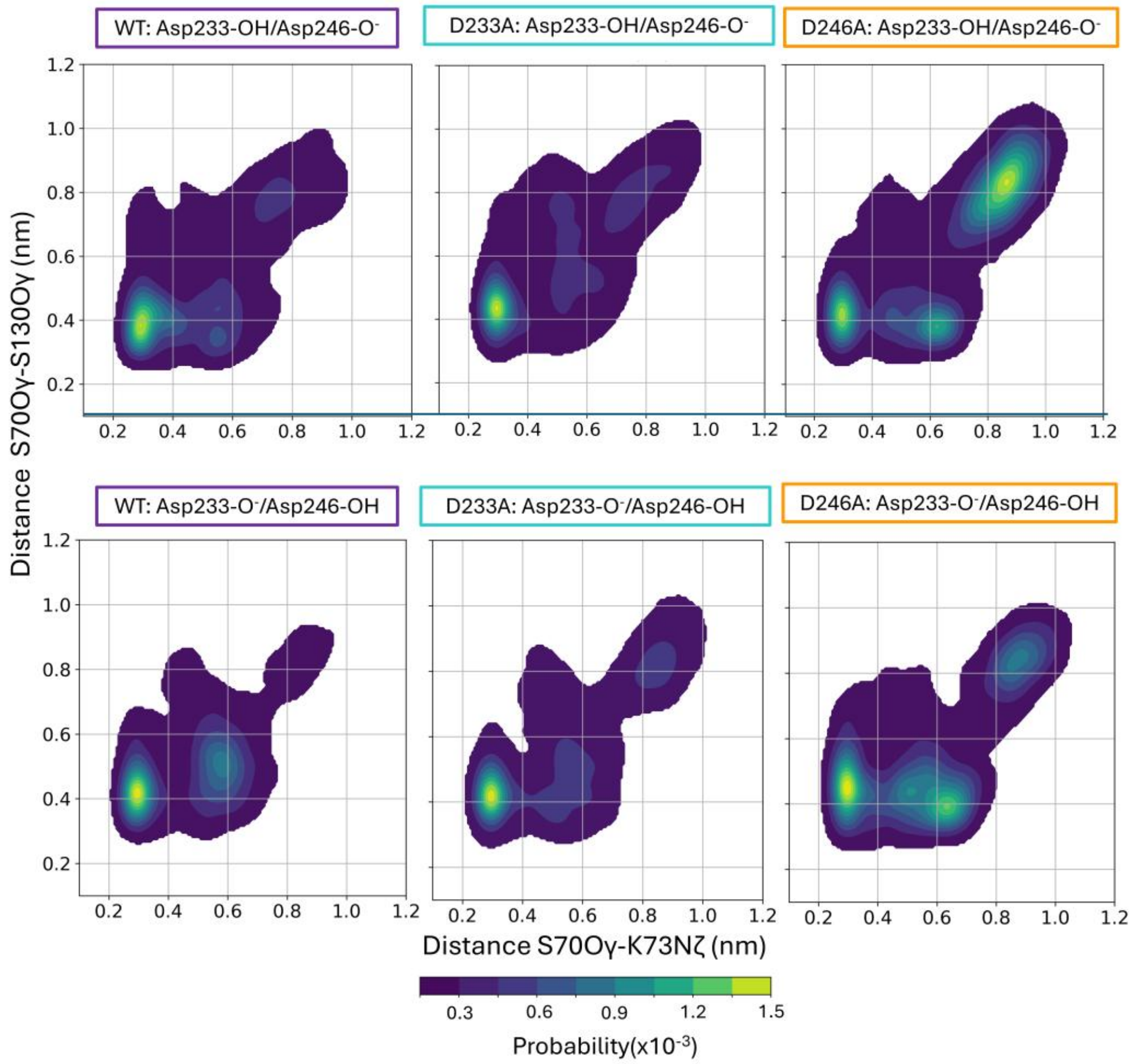

Figure S6. Correlation plots of the distances between indicated active site atoms in five replicates of 1 µs MD simulations of BlaC WT, D233A, and D246A in different protonation states. The color of the areas indicates the probability density.

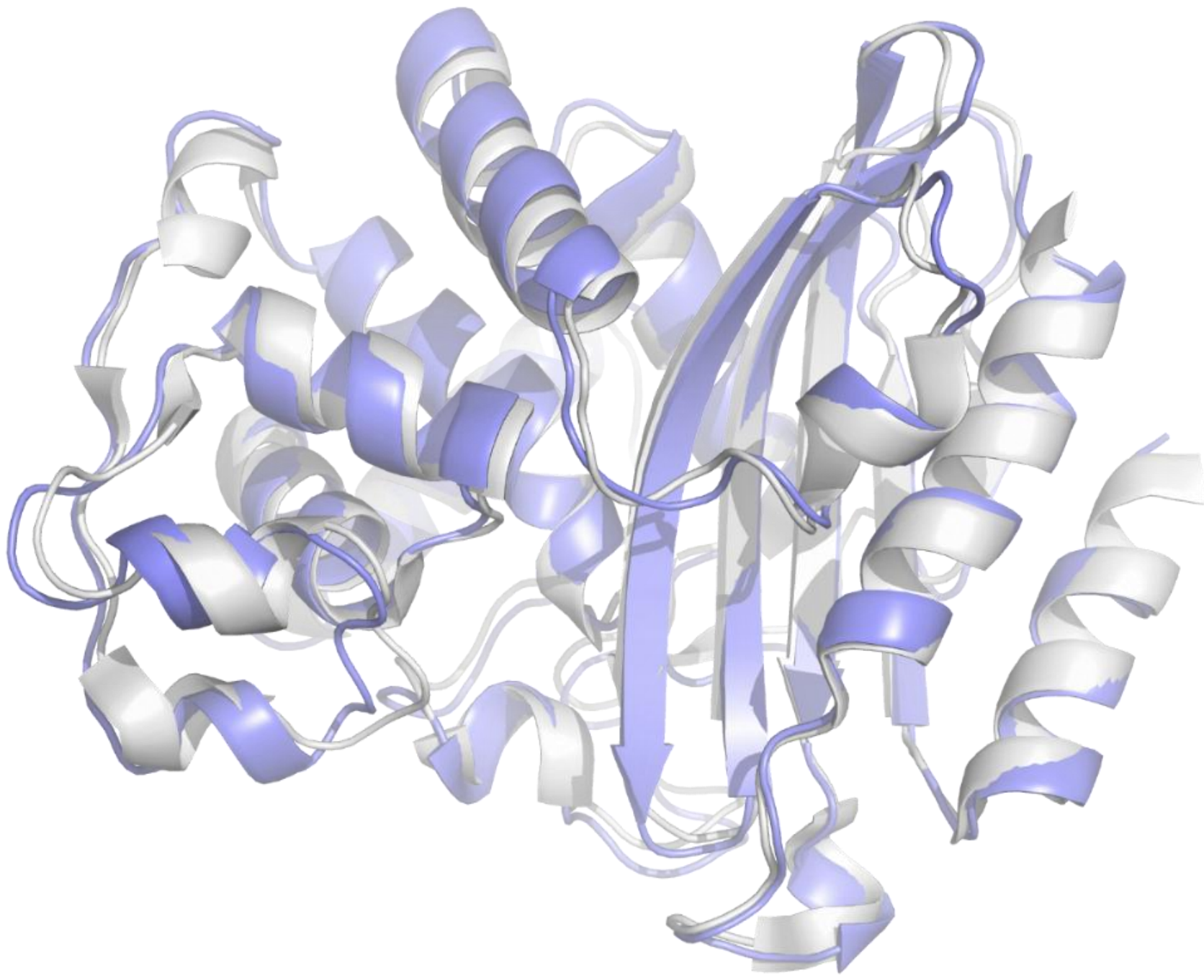

Figure S7. Structural superposition of the X-ray crystal structure pbd_00002gdn, coloured in white, and BlaC WT state A derived from molecular dynamics simulations, coloured in slate, showing an RMSD of 0.125 nm.

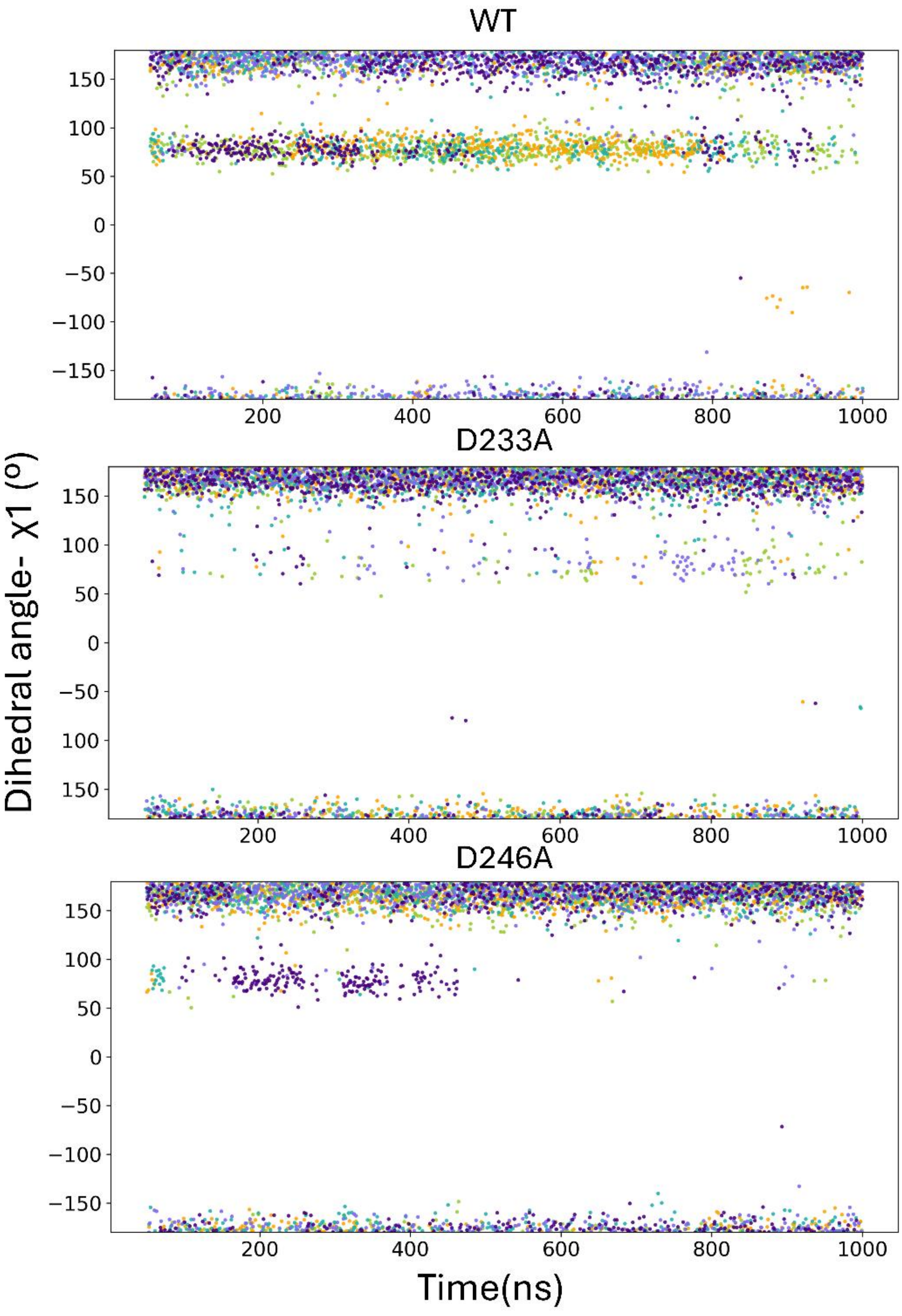

Figure S8. Dihedral angle χ_1 time traces of Ser130 in the MD simulations. Different colours represent five different replicates.

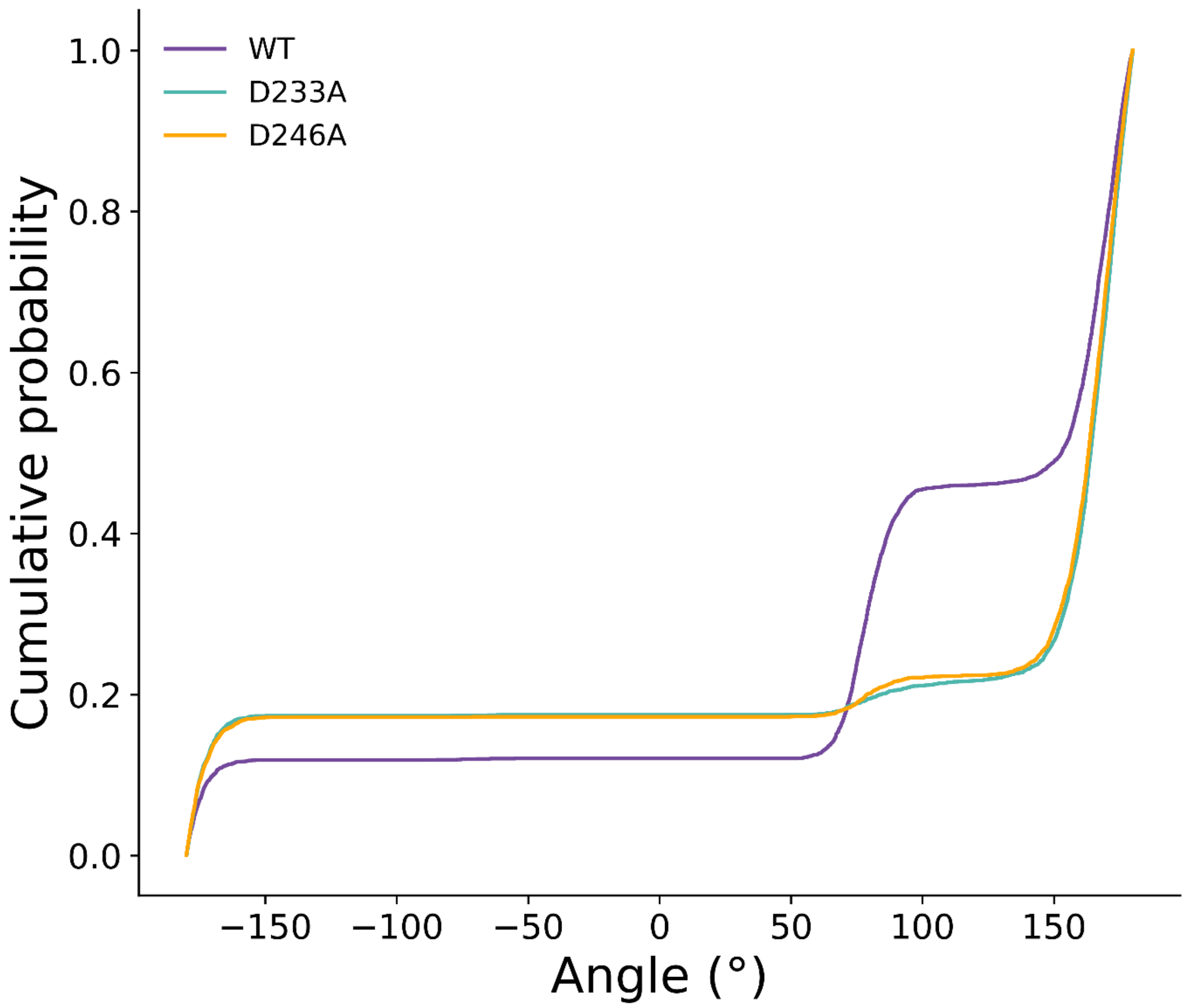

Figure S9. Kolmogorov–Smirnov (KS) test comparing the cumulative probability distributions of the Ser130 χ1 dihedral angle for WT (indigo), D233A (turquoise), and D246A (orange) across five independent replicas of each system as an illustrative example. KS statistics: WT vs. D233A, D = 0.2447 ($p \ll 0.001$); WT vs. D246A, D = 0.2374 ($p \ll 0.001$).

```
        10         20         30         40         50         60
MGSSHHHHHH SSGLVPRGSH MENLYFQSGG DLADRFAELE RRYDARLGVY VPATGTTAAI
        70         80         90        100        110        120
EYRADERFAF CSTFKAPLVA AVLHQNPLTH LDKLITYTSD DIRSISPVAQ QHVQTGMTIG
       130        140        150        160        170        180
QLCDAAIRYS DGTAANLLLA DLGGPGGGTA AFTGYLRSLG DTVSRLDAEE PELNRDPPGD
       190        200        210        220        230        240
ERDTTTPHAI ALVLQQLVLG NALPPDKRAL LTDWMARNTT GAKRIRAGFP ADWKVIDKTG
       250        260        270        280        290        300
TGDYGRANDI AVVWSPTGVP YVVAVMSDRA GGGYDAEPRE ALLAEAATCV AGVLA
```

Figure S10. The amino acid sequence of BlaC WT used for protein production. Residues corresponding to the N-terminal 6xHis tag are indicated in cyan, and the TEV protease cleavage site is highlighted in orange. Residues 31-293 correspond to residue numbers 43-307 of the BlaC Uniprot sequence P9WKD3 and are numbered according to Ambler notation. The Gly residues are present at the N-terminus (residues 29 and 30) to enhance removal of the tag by TEV.
