# Supplementary material for: Conserved Asp233–Asp246 hydrogen bond modulates active site dynamics in class A β‐lactamases
Source: Protein Sci. 2026 Sep 17;35(10):e70798. doi: 10.1002/pro.70798 (PMC13584895; doi:10.1002/pro.70798)
Supplement: Supplementary file 2 [file PRO-35-e70798-s002.pdf]

Supplementary tables to

Conserved Asp233-Asp246 Hydrogen Bond Modulates Active Site Dynamics in class A β-lactamases

Cristina V. Lopez-Gallego, Monika Timmer, Jocelyne Vreede, Marcellus Ubbink.

Table S1. Kinetic parameters of selected substrates hydrolyzed by BlaC WT, D233A, and D246A at 25 °C. The values represent the mean of triplicate measurements, and the errors are the standard deviations. For substrates for which a plateau (Figure S1) was not observed within the detection time, kinetic parameters k_{cat} and K_m^{app} could not be independently determined; instead, only the catalytic efficiency (k_{cat}/K_m^{app}) was estimated.

Substrate	Parameter	WT	D233A	D246A
Ampicillin	K_m^{app} (mM)	0.16 (0.03)	0.27 (0.04)	0.63 (0.06)
	k_{cat} (s^{-1})	12 (1)	26 (4)	10 (3)
	k_{cat}/K_m^{app} (mM^{-1} s^{-1})	72 (10)	94 (20)	28 (15)
Nitrocefin	K_m^{app} (mM)	0.147 (0.006)	0.160(0.001)	0.181 (0.002)
	k_{cat} (s^{-1})	18.6 (0.7)	15.0(0.2)	9.2 (0.2)
	k_{cat}/K_m^{app} (mM^{-1} s^{-1})	126 (9)	94 (2)	51 (1)
Cefuroxime	k_{cat}/K_m^{app} ($mM^{-1}s^{-1}$)	1.7 (0.2)	0.90 (0.22)	0.63 (0.10)
Ceftriaxone	k_{cat}/K_m^{app} ($mM^{-1}s^{-1}$)	0.63 (0.01)	0.40 (0.01)	0.23 (0.01)

Table S2. Distances mean and standard deviations for distances $d_{D172-D179}$ and $d_{D233-D246}$.

		WT	D233A	D246A
distance name	atoms	Average(nm)	Average(nm)	Average(nm)
$d_{D172-D179}$ (Asp233-OH/Asp246-O$^-$)	D172Cγ-D179Cγ	0.442(0.003)	0.442(0.001)	0.442(0.005)
$d_{D172-D179}$ (Asp233-O$^-$/Asp246-OH)	D172Cγ-D179Cγ	0.447(0.004)	0.450(0.004)	0.451(0.006)
$d_{D233-D246}$ (Asp233-OH/Asp246-O$^-$)	D233Cβ-D246Cβ	0.559(0.022), 0.695(0.020)	0.602(0.040)	0.636(0.031)
$d_{D233-D246}$ (Asp233-O$^-$/Asp246-OH)	D233Cβ-D246Cβ	0.636(0.050)	0.605(0.03)	0.645(0.042)
$d_{D233-D246}$ (Asp233-OH/Asp246-O$^-$)	D233Cγ-D246Cγ	0.421(0.008)	-	-
$d_{D233-D246}$ (Asp233-O$^-$/Asp246-OH)	D233Cγ-D246Cγ	0.429(0.019), 0.559(0.025)	-	-

Table S3. Atoms and distance range used to extract the filtered structures for structural comparison.

	Distance1		Distance2	
Range	Min	Max	Min	Max
Atom	D233Cγ	D246Cγ	-	-
Distance state 1 (S1) (WT: Asp233-OH/Asp246-O$^-$)(nm)	0.4	0.45	-	-
Distance state 1 (S1) (WT: Asp233-O$^-$/Asp246-OH)(nm)	0.4	0.45	-	-
Distance state 2 (S2) (WT: Asp233-O$^-$/Asp246-OH)(nm)	0.52	0.59	-	-
Atom	S70Oγ	K73Nζ	S70Oγ	S130Oγ
Distance state A (WT: Asp233-OH/Asp246-O$^-$)(nm)	0.25	0.35	0.36	0.43
Distance state A' (D233A and D246A: Asp233-OH/Asp246-O$^-$)(nm)	0.25	0.35	0.39	0.5
Distance state B (D246A: Asp233-OH/Asp246-O$^-$)(nm)	0.81	0.91	0.75	0.9

Table S4. Primer sequences used to introduce mutations into the *blaC* gene.

Mutation	Forward primer 5'-3'
D233A	CCG GCA GAT TGG AAA GTT ATT **GCT** AAA ACC GGT ACG GGT G
D246A	GGT GAT TAT GGT CGT GCA AAT **GCT** ATT GCA GTT GTT TGG AGC
